# Synthesis and Characterization of Process-Related Impurities of Antidiabetic Drug Linagliptin

**DOI:** 10.3390/molecules21081041

**Published:** 2016-08-09

**Authors:** Yiwen Huang, Xiaoqing He, Taizhi Wu, Fuli Zhang

**Affiliations:** Shanghai Institute of Pharmaceutical Industry, China State Institute of Pharmaceutical Industry, 285 Gebaini Road, Pudong, Shanghai 201203, China; cpuhyw2010@163.com (Y.H.); xuesitu@163.com (X.H.)

**Keywords:** linagliptin, type 2 diabetes, impurities, synthesis, characterization

## Abstract

Linagliptin, a xanthine derivative, is a highly potent, selective, long-acting and orally bioavailable DPP-4 inhibitor for the treatment of type 2 diabetes. During the process development of linagliptin, five new process-related impurities were detected by high performance liquid chromatography (HPLC). All these impurities were identified, synthesized, and subsequently characterized by their respective spectral data (MS, HRMS, ^1^H-NMR, ^13^C-NMR and IR) as described in this article. The identification of these impurities should be useful for quality control and the validation of the analytical method in the manufacture of linagliptin.

## 1. Introduction

The presence of impurities in a drug substance can have a significant impact on the quality and safety of the drug product [1]. According to the general guidelines on impurities in drug substances recommended by the International Conference on Harmonization (ICH), any impurities present in the drug substance greater than a level of 0.10% for drugs with a maximum daily dose equal to or lesser than 2 g should be identified and characterized [2]. On one hand, the identification and characterization of process-related impurities can guide us in controlling these impurities within the acceptable level by improving reaction conditions in turn. On the other hand, impurities in pure form are also needed to validate the analytical method including checking the system suitability and relative correction factor.

**Figure 1:**
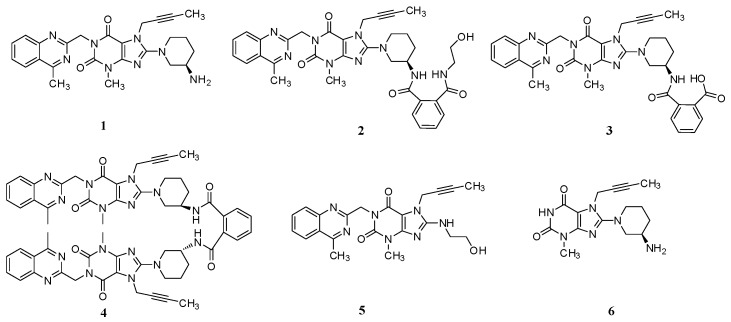
Chemical structures of linagliptin and process-related impurities.

Linagliptin (**1**, Figure 1), a highly potent, selective, long-acting and orally bioavailable DPP-4 inhibitor for the treatment of type 2 diabetes, is chemically known as (*R*)-8-(3-aminopiperidin-1-yl)-7-(but-2-yn-1-yl)-3-methyl-1-((4-methylquinazolin-2-yl)methyl)-1*H*-purine-2,6(3*H*,7*H*)-dione [3].

**Scheme 1:**
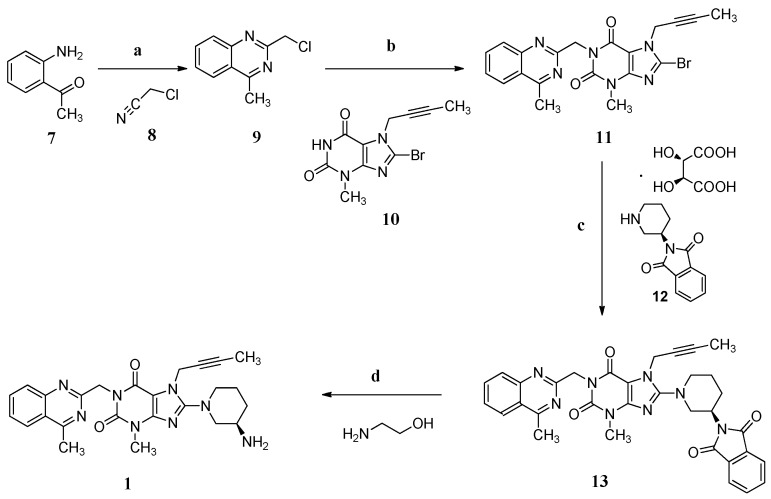
Synthesis of linagliptin (**1**). *Conditions:* (**a**) HCl, 1,4-dioxane, 6 °C , 2 h, 74%–85%; (**b**) Na_2_CO_3_, *N*-methyl-2-pyrrolidone (NMP), 140 °C, 2 h, 76%–83%; (**c**) diisopropanolamine, NMP, 140 °C, 2 h, 90%–94%; (**d**) ethanolamine, THF/H_2_O, 60 °C, 3 h, 81.9%.

In the industrial manufacturing process (Scheme 1) of linagliptin by the Boehringer Ingelheim company [4], cyclization of 1-(2-aminophenyl)ethanone (**7**) with 2-chloroacetonitrile (**8**) in the presence of hydrogen chloride afforded 2-(chloromethyl)-4-methylqu inazoline (**9**, yield 74%–85%) which condensed with 8-bromo-7-(but-2-yn-1-yl)-3-methyl-1*H*-purine-2,6(3*H*,7*H*)-dione (**10**) in the presence of sodium carbonate as a basic reagent, giving 8-bromo-7-(but-2-yn-1-yl)-3-methyl-1-((4-methylquinazolin-2-yl)methyl)-1*H*-puri ne-2,6(3*H*,7*H*)-dione (**11**, yield 76%–83%). Subsequently, the condensation of **11** with (*R*)-2-(piperidin-3-yl)isoindoline-1,3-dione D-(−)-tartaric acid (**12**) using diisopropylethylamine as a basic reagent provided (*R*)-7-(but-2-yn-1-yl)-8-(3-(1,3-dioxoisoindolin-2-yl)pipe ridin-1-yl)-3-methyl-1-((4-methylquinazolin-2-yl)methyl)-1*H*-purine-2,6(3*H*,7*H*)-dione (**13**, yield 90%–94%). Compound **13** finally converted to the desired linagliptin (**1**) in 81.9% yield via aminolysis using ethanolamine as the aminolysis agent.

However, the literature search did not reveal much work on the impurity research of linagliptin. The impurity profile of linagliptin in this synthetic route is different from the earlier reported study [5,6], which makes it more challenging to identify the unknown impurities formed in small quantities in the drug substance. Since most of the time it is very difficult to identify and control impurities within acceptable levels in the process, some advanced purification techniques for an active drug substance may then be taken into account, such as the continuous-flow process [7], organic solvent nanofiltration (OSN) [8], molecularly imprinted membranes for OSN [9] and counter current chromatography (CCC), as a valuable addition to the chromatography toolbox [10].

Hence, a comprehensive study was undertaken to identify and synthesize the impurities in a sample of linagliptin as described in this article. The study will help to understand the formation of the impurities in linagliptin synthesis and provide a clue on how to obtain a pure drug substance.

## 2. Results and Discussion

### 2.1. Structures of Impurities

**Figure 2:**
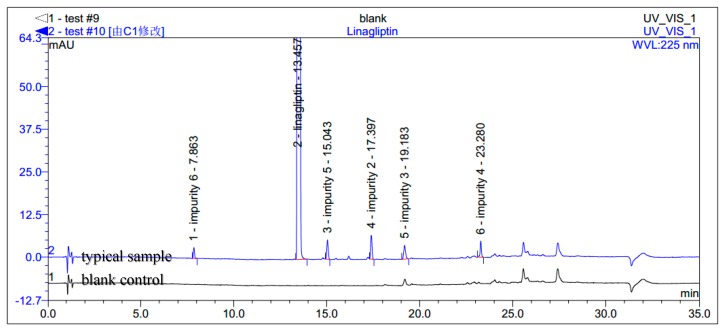
Typical HPLC chromatogram of linagliptin with impurities.

During the process development of linagliptin, five new process-related impurities were detected by high performance liquid chromatography (HPLC) (Figure 2), varying from 0.15% to 0.5%, and the molecular weight of the respective impurities was identified through liquid chromatographic mass spectrometry (LC-MS). From the molecular weight information, an extensive study was undertaken to predict and synthesize the five new impurities. Finally, all these impurities were synthesized and subsequently subjected to spectral analysis. The five predicted and synthesized impurities showed the same retention time with those detected in HPLC. Based on the spectral data, these impurities were characterized as the structures shown in Figure 1. The five new impurities are all reported here for the first time.

### 2.2. Source of Impurities

**Scheme 2:**
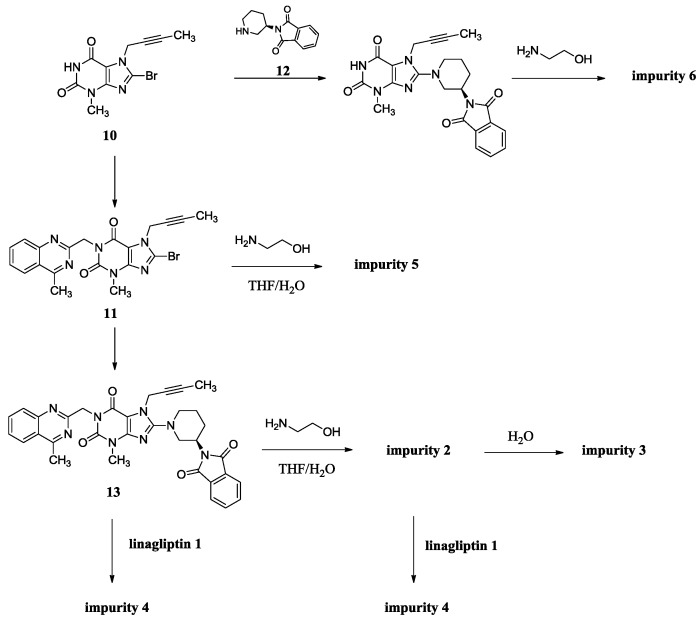
Source of impurities of linagliptin.

The synthesis of linagliptin involved the aminolysis of **13** using 10 equivalents of ethanolamine [4]. Compound **2** is the by-product in this step, formed from incomplete aminolysis. Impurity **2** was further hydrolyzed to afford impurity **3** due to water/tetrahydrofuran (THF) as a solvent. The formed linagliptin moiety (**1**) reacted with starting material **13** or with **2**, providing impurity **4**. During the condensation of **11** with **12** to afford **13**, the intermediate **11** cannot be converted completely and may remain in the intermediate **13**. Unfortunately, the small amount of **11** remaining in the intermediate **13** reacted with ethanolamine in the next aminolysis process, which explains the formation of impurity **5**. Similarly, during the condensation of **10** with **9** to afford **11**, the intermediate **10** cannot be converted completely and may remain in the intermediate **11**. Subsequently, **10** remaining in the intermediate **11** took part in the condensation reaction with **12** and in the aminolysis reaction in the next two steps, which finally leads to the formation of impurity **6** (Scheme 2). As a result, the five impurities accounted for 0.4%, 0.32%, 0.27%, 0.33% and 0.16%, respectively, in the drug substance.

### 2.3. Preparation and Characterization of Impurities

**Scheme 3:**
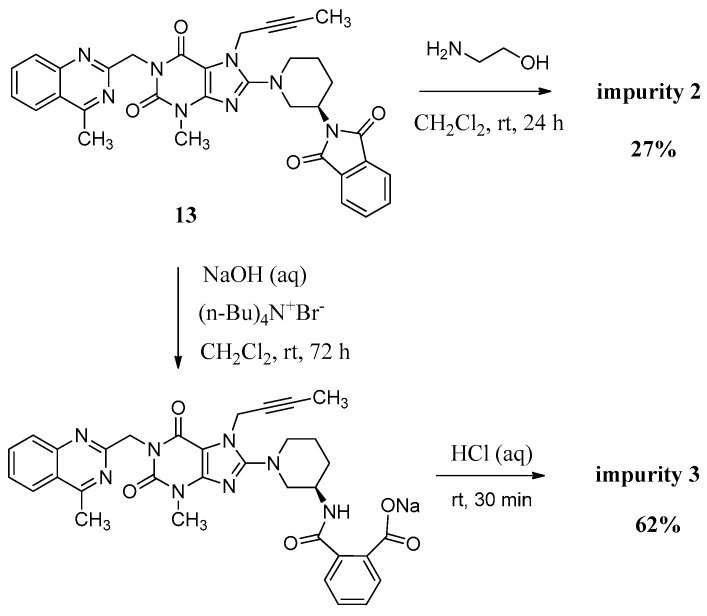
Preparation of impurity **2** and **3**.

Compound **2** was synthesized by incomplete aminolysis of **13**. In order to prepare **2** in good yield, we replaced the solvent of THF/H_2_O with dichloromethane (CH_2_Cl_2_) to obtain a homogeneous system and a smaller inventory ratio of ethanolamine was used for more moderate aminolysis conditions (Scheme 3).

The mass spectrum of **2** showed a molecular ion peak at *m/z* 686.23 (M + Na)^+^ in positive ion mode, indicating the mass of this compound to be 663. The ^1^H-NMR of this compound revealed three additional D_2_O exchangeable signals at 8.35, 6.95 and 3.70, indicating the two amide N-H protons and one O-H proton. Additional signals observed in the region of 8.04–7.47 indicated eight aromatic C-H protons. In the IR spectrum, observed bands at 3473 and 3263 cm^−1^ indicated the amide N-H and O-H stretching. The ^13^C-NMR revealed 35 carbon atoms and further Distortionless Enhancement by Polarization Transfer (DEPT) exhibited eight secondary carbon atoms. Based on all the spectral data (MS, HRMS, ^1^H-NMR, ^13^C-NMR and IR), the structure of impurity **2** was confirmed as (*R*)-*N*^1^-(1-(7-(but-2-yn-1-yl)-3-methyl-1-((4-methylquinazolin-2-yl)methyl)-2,6-dioxo-2,3,6,7-tetrahydro-1*H*-purin-8-yl)piperidin-3-yl)-*N*^2^-(2-hydroxyethyl)phthalamide.

Compound **3** was prepared by the alkali hydrolysis of **13** with phase transfer catalyst (Scheme 3). The undesired product **1** from excessive hydrolysis can be removed by CH_2_Cl_2_.

**Figure 3:**
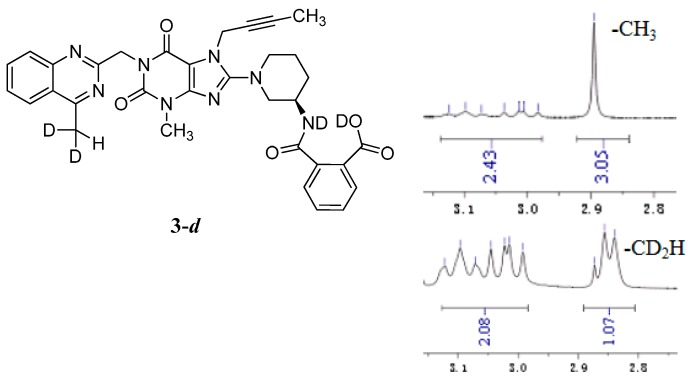
Structures and partial ^1^H-NMR spectrum of **3-*d***.

The mass spectrum of **3** exhibited a molecular ion peak at *m/z* 643.25 (M + Na)^+^ in positive ion mode and *m/z* 619.31 (M − H)^−^ in negative ion mode, indicating the mass of this compound to be 620. The ^1^H-NMR of this impurity revealed two D_2_O exchangeable signals at 12.86 and 8.35, indicating one acid O-H proton and one amide N-H proton, and an additional eight proton signals at 7.41–8.25 corresponding to aromatic protons. Interestingly, we found the methyl group (CH_3_) in quinazoline at δ 2.89 ppm is acidic at a certain degree, which explains the proton exchange of the CH_3_ group with D_2_O which takes place sometimes. The proton exchange’s resulting compound (**3-*d***, Figure 3) also explains the proton splitting of the CH_3_ group, which showed a triplet peak with one proton of 2.89 ppm in the ^1^H-NMR with D_2_O added (Figure 3). The IR spectrum of **3** exhibited a band at 3527 cm^−1^ corresponding to amide N-H stretching, and 3255 cm^−1^ for acid O-H stretching. The ^13^C-NMR revealed 33 carbon atoms and further DEPT showed six secondary carbon atoms. Impurity **3** was characterized by all respective spectral data (MS, HRMS, ^1^H-NMR, ^13^C-NMR and IR) as (*R*)-2-((1-(7-(but-2-yn-1-yl)-3-methyl-1-((4-methylquinazolin-2-yl)methyl)-2,6-dioxo-2,3,6,7-tetrahydro-1*H*-purin-8-yl)piperidin-3-yl)carbamoyl)benzoic acid.

**Scheme 4:**
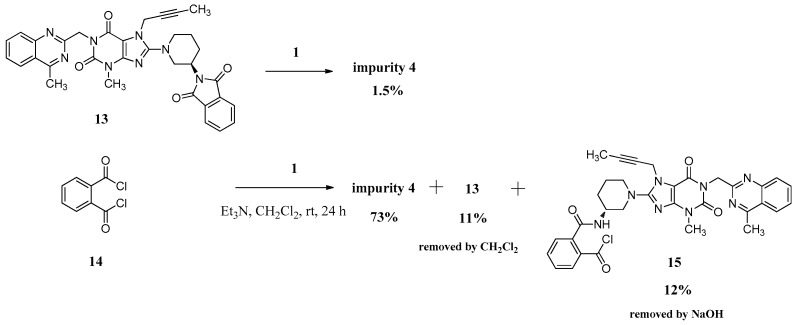
Preparation of impurity **4**.

We can only obtain compound **4** with the low yield of 1.5% directly via **13** reacting with the linagliptin moiety (**1**). Instead, we have exploited a different concept for the synthesis of compound **4** starting from phthaloyl dichloride (**14**). However, 12% of the single-benzoyl chloride compound **15** and 11% of the cyclization product **13** would be generated meanwhile due to the steric hindrance from linagliptin, which influenced the purity of **4**. We reduced them to below 0.3% and finally got compound **4** with a high purity of 99.15% by washing the reaction mass with CH_2_Cl_2_ and aqueous sodium hydroxide solution instead of the column chromatography (Scheme 4).

The mass spectrum of **4** exhibited a molecular ion peak at *m/z* 1075.24 (M + H)^+^, 1097.22 (M + Na)^+^ in positive ion mode, indicating the mass of this compound to be 1074. The molecular formula of this compound was C_58_H_58_N_16_O_6_ as confirmed by the high resolution mass spectrum (HRMS). In the IR spectrum the observed band at 3243 cm^−1^ indicated the amide N-H. The ^13^C-NMR showing 29 carbon atoms indicated the symmetry in the structure of compound **4**. Based on all the spectral data (MS, HRMS, ^1^H-NMR, ^13^C-NMR and IR), the structure of impurity **4** was confirmed as *N*,*N*-bis((*R*)-1-(7-(but-2-yn-1-yl)-3-methyl-1-((4-methylquinazolin-2-yl)methyl)-2,6-dioxo-2,3,6,7-tetrahydro-1*H*-purin-8-yl)piperidin-3-yl)phthalamide.

**Scheme 5:**
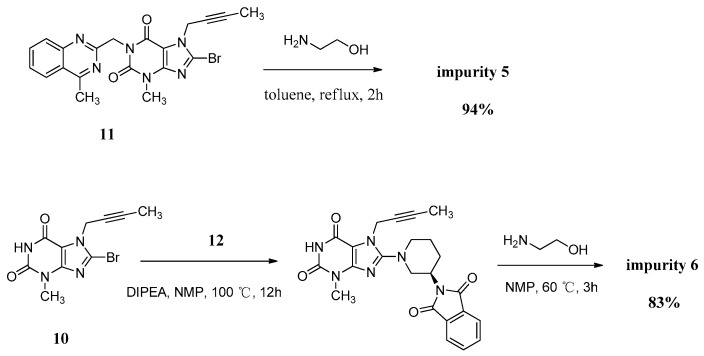
Preparation of impurities **5** and **6**.

Compound **5** was independently provided via the condensation of compound **11** with ethanolamine. In order to prepare **5** in good yield, we replaced the solvent of tetrahydrofuran/water with toluene to obtain a homogeneous system and a more violent reaction temperature. The increasing inventory ratio of ethanolamine also contributed to the high yield of 94% (Scheme 5).

The mass spectrum of **5** showed a molecular ion peak at *m/z* 456.51 (M + Na)^+^ in positive ion mode, indicating the mass of this compound to be 433. The ^1^H-NMR of this compound revealed two D_2_O exchangeable signals at 7.23 and 4.78, indicating the N-H and O-H protons, and an additional four proton signals at 7.62–8.25 corresponding to aromatic protons. In the IR spectrum the observed bands at 3446 and 3364 cm^−1^ indicated the N-H and O-H stretching. The ^13^C-NMR revealed 22 carbon atoms and DEPT showed four secondary carbon atoms. Based on all the spectral data (MS, HRMS, ^1^H-NMR, ^13^C-NMR and IR), the structure of impurity **5** was confirmed as 7-(but-2-yn-1-yl)-8-((2-hydroxyethyl)amino)-3-methyl-1-((4-methylquinazolin-2-yl)methyl)-1*H*-purine-2,6(3*H*,7*H*)-dione.

Compound **6** was independently synthesized, starting from compound **10**, following a synthetic process analogous to that of linagliptin. Differently, we have exploited a one-pot process for the synthesis of compound **6** instead of the two-step operation in greater yield (Scheme 5).

The mass spectrum of **6** showed a molecular ion peak at m/z 317.14 (M + H)^+^ in positive ion mode, indicating the mass of this compound to be 316, which is 156 amu less than that of linagliptin (**1**). The ^1^H-NMR of this compound revealed two D_2_O exchangeable signals at 10.94 and 8.27, indicating the imide N-H proton and N-H_2_ proton, and no additional aromatic protons were observed. In the IR spectrum the observed band at 3021 cm^−1^ indicated the imide N-H stretching and double peaks of 3115 and 3074 cm^−1^, corresponding to primary amine N-H stretching. The ^13^C-NMR revealed 15 carbon atoms and DEPT showed five secondary carbon atoms. Impurity **6** was characterized by all respective spectral data (MS, HRMS, ^1^H-NMR, ^13^C-NMR and IR) as (*R*)-8-(3-aminopiperidin-1-yl)-7-(but-2-yn-1-yl)-3-methyl-1*H*-purine-2,6(3*H*,7*H*)-dione.

## 3. Experimental Section

### General Information

The compound **11** (chemical purity 98.36%), **13** (chemical purity 97.92%; chiral purity 99.97%) and **1** (chemical purity 98.35%; chiral purity 99.97%) were prepared according to the literature procedure [4]. Other materials, solvents and reagents were of commercial origin and used without additional operations.

The ^1^H- and ^13^C-NMR spectra were recorded on a Bruker Arance III 400 MHz spectrometer. The solvents used were DMSO-*d*_6_ or CDCl_3_. The ^1^H-NMR chemical shift values were reported as δ ppm relative to tetramethylsilane (TMS) and the ^13^C-NMR chemical shift values were reported on δ ppm relative to DMSO-*d*_6_ or CDCl_3_. DEPT spectra revealed the presence of methyl and methine groups as positive peaks and methylene as negative peaks. The IR spectra were recorded in the solid state as KBr dispersion using a NICOLET 670 FT-IR spectrophotometer. Mass spectra and high resolution mass spectrum were recorded on Agilent 6120B series single quadrupole LC-MS and Q-Tof micro YA019 instrument. Melting points were measured on a WRS-1B apparatus. The specific rotation was calculated from an optical rotation measurement performed on the Autopol IV, serial number 80799 (Rudolph, Hackettstown, NJ, USA) at the wavelength of 589 nm (D line of a sodium lamp), at 20 °C.

The HPLC analyses were recorded on a Dionex UItiMate 3000 HPLC instrument using Agilent Eclipse XDB C18 column (150 mm × 4.6 mm, 5 µm) in a thermostated column heater at 55 °C. The mobile phases consisting of A (0.1% methanoic acid, pH 2.5) and B (acetonitrile) were used with the gradient mode at the flow rate of 1.5 mL/min. The UV detection at 225 nm was used. Initial gradient starts with 5% of B and at 18 min it was set to 40%. The ratio had been set to 70% at 30 min and at 30.1 min it was 5%, which continued up to 35 min. The samples were diluted in acetonitrile with a concentration of 0.5 mg/mL. The injection volume was 5 µL. Limit of Detection (LOD) was 0.10 μg/mL or 0.02% for impurities **2**, **3**, **4**, **5** and 0.15 μg/mL or 0.03% for impurity **6**. Limit of Quantity (LOQ) was 0.25 μg/mL or 0.05% for impurities **2**, **3**, **4**, **5** and 0.33 μg/mL or 0.07% for impurity **6**.

This HPLC method further subjected for LC-MS. Samples were run in Electro-Spray Ionization positive mode (ESI+) and 12 L/min nebulizer gas flow rate. The fragmentor voltage was 70 V and capillary voltage was maintained at 3.0 kV. The drying gas temperature was at 350 °C.

The detailed spectral data (IR, ^1^H-NMR, ^13^C-NMR, MS, HRMS) of the compounds **2**–**6** as well as the HPLC chromatogram of the compounds **1**–**6** are provided in the Supplementary Materials.

*(R)-N^1^-(1-(7-(But-2-yn-1-yl)-3-methyl-1-((4-methylquinazolin-2-yl)methyl)-2,6-dioxo-2,3,6,7-tetrahydro-1H-purin-8-yl)piperidin-3-yl)-N^2^-(2-hydroxyethyl)phthalamide (***2***)*. To a stirred solution of compound **13** (5.0 g, 0.008 mol) in CH_2_Cl_2_ (50.0 mL) was added ethanolamine (1.5 g, 0.024 mol) and maintained the reaction mass at room temperature for 24 h. Then added 15 mL H_2_O stirring for 30 min to dilute ethanolamine. The isolated solid was collected by filtration and washed with 10 mL H_2_O and 20 mL CH_2_Cl_2_. Then dried to yield **2** as a light yellow solid (1.5 g, 27.3%), HPLC purity 97.52%; m.p. 189–192 °C; ${\left[\alpha \right]}_{D}^{20}$ −32.567 (*c* = 1 g/100 mL, DMSO); IR (KBr) ν_max_ 3473, 3263 (amide N-H_ν_, O-H_ν_); 3062 (aromatic C-H_ν_); 2937, 2850 (C-H_ν_); 2230 (C≡C_ν_); 1698, 1662 (C=O_ν_); 1522 (aromatic C=C_ν_, aromatic C=N_ν_); 1440, 1400 (C-H_δ_); 1228 (C-O_ν_); 763 (aromatic C-H_γ_) cm^−1^; ^1^H-NMR (400 MHz, CDCl_3_) δ 8.35 (d, *J* = 6.6 Hz, 1H), 8.04 (d, *J* = 8.2 Hz, 1H), 7.89 (d, *J* = 8.4 Hz, 1H), 7.82–7.76 (m, 1H), 7.64–7.52 (m, 3H), 7.47 (m, 2H), 6.95 (t, *J* = 5.6 Hz, 1H), 5.56 (s, 2H), 4.89 (q, *J* = 2.0 Hz, 2H), 4.31 (s, 1H), 3.78 (m, 2H), 3.70 (m, 2H), 3.60 (m, 2H), 3.52 (m, 2H), 3.40 (m, 1H), 3.23 (s, 3H), 2.91 (s, 3H), 2.17–2.10 (m, 1H), 1.92 (m, 2H), 1.75 (not resolved, 4H); ^13^C-NMR (100 MHz, DMSO-*d*_6_) δ 169.29, 168.75, 168.46, 161.44, 156.37, 153.80, 151.41, 149.51, 148.03, 136.58, 136.51, 134.51, 129.80, 129.67, 128.33, 128.26, 128.02, 127.57, 126.13, 122.94, 103.90, 81.74, 74.21, 60.22, 53.84, 50.47, 46.13, 46.08, 42.55, 35.90, 29.90, 29.59, 23.53, 22.01, 3.55; HRMS (ESI) *m/z* 664.3019 (calcd for C_35_H_38_N_9_O_5_, 664.2996 [M + H]^+^).

*(R)-2-((1-(7-(But-2-yn-1-yl)-3-methyl-1-((4-methylquinazolin-2-yl)methyl)-2,6-dioxo-2,3,6,7-tetrahydro-1H-purin-8-yl)piperidin-3-yl)carbamoyl)benzoic acid (***3***)*. To a stirred solution of compound **13** (5.0 g, 0.008 mol) in CH_2_Cl_2_ (50.0 mL) was added aqueous sodium hydroxide solution (1.0 g of NaOH in 50.0 mL of H_2_O) and tetra-n-butylammonium bromide (0.27 g, 0.0008 mol). Then maintained the reaction mass at rt for 72 h. Then water phase was collected and washed with CH_2_Cl_2_ (25.0 mL) for three times. To the stirred water phase was added 1 mol/L aq. HCl (25.0 mL) and maintained at rt for 30 min. The isolated solid was collected by filtration and washed with water (20.0 mL) and CH_2_Cl_2_ (20.0 mL). Then dried to yield **3** as a light yellow solid (3.2 g, 62.2%), HPLC purity 97.34%; m.p. 144–148 °C; ${\left[\alpha \right]}_{D}^{20}$ −49.700 (*c* = 1 g/100 mL, DMSO); IR (KBr) ν_max_: 3527 (amide N-H_ν_); 3255 (acid O-H_ν_); 3068 (aromatic C-H_ν_); 2944, 2856 (C-H_ν_); 2224 (C≡C_ν_); 1700, 1654 (C=O_ν_); 1573, 1519 (aromatic C=C_ν_, aromatic C=N_ν_); 1440, 1400 (C-H_δ_); 763 (aromatic C-H_γ_) cm^−1^; ^1^H-NMR (400 MHz, DMSO-*d*_6_) δ 12.86(s, 1H), 8.35 (d, *J* = 7.8 Hz, 1H), 8.25 (d, *J* = 8.2 Hz, 1H), 7.96–7.89 (m, 1H), 7.85–7.78 (m, 2H), 7.68 (t, *J* = 7.6 Hz, 1H), 7.59 (td, *J* = 7.5, 1.1 Hz, 1H), 7.51 (td, *J* = 7.6, 1.1 Hz, 1H), 7.41 (d, *J* = 7.4 Hz, 1H), 5.34 (s, 2H), 4.92 (q, *J* = 2.1 Hz, 2H), 4.10–4.01 (m, 1H), 3.82 (m, 1H), 3.63 (m, 1H), 3.40 (s, not resolved, CH_3_ and water), 3.14–2.98 (m, 2H), 2.89 (s, 3H), 2.02–1.86 (m, 2H), 1.76 (not resolved, 4H), 1.59 (m, 1H); ^13^C-NMR (100 MHz, DMSO-*d*_6_) δ 169.44, 169.28, 168.79, 161.43, 156.46, 153.79, 151.40, 149.50, 148.06, 137.82, 134.51, 133.49, 130.68, 129.64, 129.51, 128.35, 128.32, 127.57, 126.12, 122.93, 103.88, 81.70, 74.26, 53.98, 50.43, 46.06, 46.01, 35.92, 29.91, 29.56, 23.56, 22.01, 3.56; HRMS (ESI) *m/z* 621.2599 (calcd for C_33_H_33_N_8_O_5_, 621.2574 [M + H]^+^).

*N,N-bis((R)-1-(7-(But-2-yn-1-yl)-3-methyl-1-((4-methylquinazolin-2-yl)methyl)-2,6-dioxo-2,3,6,7-tetrahydro-1H-purin-8-yl)piperidin-3-yl)phthalamide (***4***)*. Phthaloyl dichloride (1.0 g, 0.005 mol) was added slowly to a stirred solution of **1** (4.8 g, 0.010 mol) and triethylamine (5.0 g, 0.05 mol) in CH_2_Cl_2_ (50.0 mL) at rt and refluxed for 24 h. Then the mixture was cooled to rt and 1 mol/L aq. NaOH (30 mL) was added, and maintained for 2 h. The isolated solid was collected by filtration and washed with water (20.0 mL), CH_2_Cl_2_ (20.0 mL) and methanol (20.0 mL). Then dried to yield **4** as a light yellow solid (3.5 g, 66.1%), HPLC purity 99.15%; ${\left[\alpha \right]}_{D}^{20}$ = −18.133 (*c* = 1 g/100 mL, CHCl_3_); IR (KBr) ν_max_ 3243 (amide N-H_ν_); 3068 (aromatic C-H_ν_); 2945, 2858 (C-H_ν_); 2224 (C≡C_ν_); 1700, 1662 (C=O_ν_); 1634, 1563, 1519 (aromatic C=C_ν_, aromatic C=N_ν_); 1440, 1400 (C-H_δ_); 763 (aromatic C-H_γ_) cm^−1^; ^1^H-NMR (400 MHz, DMSO-*d*_6_) δ 8.37 (d, *J* = 7.6 Hz, 1H), 8.22 (d, *J* = 8.1 Hz, 1H), 7.92–7.85 (m, 1H), 7.79 (d, *J* = 8.3 Hz, 1H), 7.67–7.62 (m, 1H), 7.50 (m, 2H), 5.30 (s, 2H), 4.90 (s, 2H), 4.10–4.00 (m, 1H), 3.81 (d, *J* = 9.0 Hz, 1H), 3.65 (m, 1H), 3.36 (s, 3H), 3.15–2.99 (m, 2H), 2.88 (s, 3H), 1.98–1.84 (m, 2H), 1.74 (not resolved, 4H), 1.65–1.55 (m, 1H); ^13^C-NMR (100 MHz, CDCl_3_) δ 168.68, 168.52, 161.01, 155.73, 154.36, 151.71, 149.89, 147.29, 135.04, 133.23, 130.18, 128.80, 128.38, 126.71, 124.83, 123.10, 104.60, 81.53, 73.01, 53.57, 51.11, 46.32, 45.83, 35.63, 29.54, 28.76, 21.74, 21.67, 3.60; HRMS (ESI) *m/z* 1075.4800 (calcd for C_58_H_59_N_16_O_6_, 1075.4803 [M + H]^+^).

*7-(But-2-yn-1-yl)-8-((2-hydroxyethyl)amino)-3-methyl-1-((4-methylquinazolin-2-yl)methyl)-1H-purine-2,6(3H,7H)-dione (***5***)*. Ethanolamine (8.0 g, 0.13 mol) was added to a stirred solution of **11** (2.0 g, 0.0044 mol) in toluene (32.0 mL) at reflux temperature and maintained for 2 h. Then the mixture was cooled to rt and 20 mL H_2_O was added, and stirred for 30 min. The isolated solid was collected by filtration and washed with toluene (10.0 mL). Then dried to yield **5** as a white solid (1.8 g, 94.2%), HPLC purity 99.61%; m.p. 238–239 °C; IR (KBr) ν_max_ 3446, 3364 (N-H_ν_, O-H_ν_); 2936, 2867 (C-H_ν_); 1702, 1652 (C=O_ν_); 1619, 1581, 1540 (aromatic C=C_ν_, aromatic C=N_ν_); 1448, 1397 (C-H_δ_); 1225 (C-O_ν_); 764 (aromatic C-H_γ_) cm^−1^; ^1^H-NMR (400 MHz, DMSO-*d*_6_) δ 8.25 (d, *J* = 8.0 Hz, 1H), 7.95–7.88 (m, 1H), 7.82 (d, *J* = 8.2 Hz, 1H), 7.72–7.62 (m, 1H), 7.23 (t, *J* = 5.6 Hz, 1H), 5.31 (s, 2H), 4.89 (q, *J* = 2.2 Hz, 2H), 4.78 (t, *J* = 5.5 Hz, 1H), 3.61 (t, *J* = 6.0 Hz, 2H), 3.46 (t, *J* = 6.0 Hz, 2H), 3.39 (s, 3H), 2.89 (s, 3H), 1.77 (t, *J* = 2.1 Hz, 3H); ^13^C-NMR (100 MHz, DMSO-*d*_6_) δ 169.19, 161.68, 154.38, 152.99, 151.50, 149.54, 149.52, 134.45, 128.31, 127.51, 126.11, 122.92, 101.31, 81.06, 74.27, 60.20, 45.89, 45.56, 33.13, 29.80, 21.99, 3.57; HRMS (ESI) m/z 434.1937 (calcd for C_22_H_24_N_7_O_3_, 434.1941 [M+H]^+^).

*(R)-8-(3-Aminopiperidin-1-yl)-7-(but-2-yn-1-yl)-3-methyl-1H-purine-2,6(3H,7H)-dione (***6***).* Diisopropylethylamine (9.8 g, 0.076 mol) was added to a stirred solution of **10** (5.0 g, 0.017 mol) and **12** (9.6 g, 0.025 mol) in NMP (50 mL) and maintained the reaction mass at 100 °C for 13 h. Then ethanolamine (10.2 g, 0.17 mol) was added at 65 °C and stirred for 4 h at 65 °C. The isolated solid was collected by filtration and washed with NMP (30 mL). Then dried to yield **6** as a off-white solid (4.4g, 82.7%), HPLC purity 99.36%; m.p. 299–302 °C; ${\left[\alpha \right]}_{D}^{20}$ −3.467 (*c* = 1 g/100 mL, DMSO); IR (KBr) ν_max_ 3115, 3074 (primary amine N-H_ν_); 3020 (imide N-H_ν_); 2947, 2793 (C-H_ν_); 2242 (C≡C_ν_); 1706 (C=O_ν_); 1660 (C=O_ν_, primary amine N-H_δ_); 1610, 1519 (C=C_ν_, C=N_ν_); 1445, 1384 (C-H_δ_) cm^−1^; ^1^H-NMR (400 MHz, DMSO-*d*_6_) δ 10.94 (s, 1H), 8.27 (s, 2H), 4.92 (q, *J* = 2.4 Hz, 2H), 3.65 (m, 1H), 3.48–3.40 (m, 1H), 3.33 (incompletely resolved, 4H), 3.15 (m, 2H), 2.00 (m, 1H), 1.96–1.85 (m, 1H), 1.81 (t, *J* = 2.2 Hz, 3H), 1.67 (m, 2H); ^13^C-NMR (100 MHz, DMSO-*d*_6_) δ 154.97, 153.99, 150.76, 148.37, 103.93, 81.20, 73.75, 51.43, 50.33, 46.09, 35.16, 28.49, 27.27, 21.85, 3.14; HRMS (ESI) *m/z* 317.1714 (calcd for C_15_H_20_N_6_O_2_, 317.1721 [M + H]^+^).

## 4. Conclusions

Five new process-related impurities detected by HPLC varying from 0.15% to 0.5% were identified, synthesized, and subsequently characterized by HPLC, MS, HRMS, ^1^H-NMR, ^13^C-NMR (DEPT) and IR techniques.

We have developed appropriate in-process checks and strategies in order to control these impurities within the acceptable level. For example, impurities **3**, **4** and **5** could be reduced to below the identification threshold of 0.1% by salifying linagliptin with hydrochloric acid while impurities **2** and **6** could be controlled within 0.1% by recrystallization from toluene. The detection limit of impurities **2**, **3**, **4** and **5** was 0.10 μg/mL or 0.02% while the detection limit of impurity **6** was 0.15 μg/mL or 0.03%. Our efforts to synthesize and characterize them effectively prove to be valuable when it comes to complying with the regulatory norms as well as assessing the quality of linagliptin.

## Supplementary Materials

Supplementary materials can be accessed at: http://www.mdpi.com/1420-3049/21/8/1041/s1.
