# Supplementary material for: Synthesis and Characterization of Process-Related Impurities of Antidiabetic Drug Linagliptin
Source: Molecules. 2016 Aug 9;21(8):1041. doi: 10.3390/molecules21081041 (PMC6274017; doi:10.3390/molecules21081041)
Supplement: Supplementary file 1 [file molecules-21-01041-s001.pdf]

Supplementary Material: Synthesis and Characterization of Process-Related Impurities of Antidiabetic Drug Linagliptin

Yiwen Huang, Xiaoqing He, Taizhi Wu, and Fuli Zhang

10 Linagliptin

样品名:	Linagliptin	*进样量:*	5.0
瓶序号:	GC3	*通道:*	UV_VIS_1
样品类型:	unknown	*波长:*	225.0
控制程序:	利格列汀N-008	*带宽:*	4
定量方法:	利格列汀	*稀释因子:*	1.0000
记录时间:	2016/6/15 16:37	*样品重量:*	1.0000
运行时间 (min):	35.00	*样品量:*	1.0000

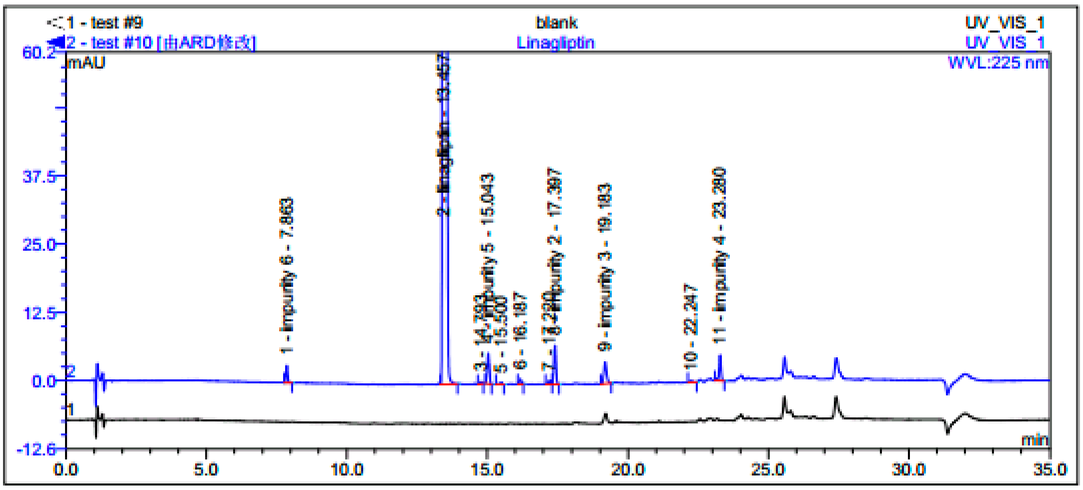

序号	保留时间 min	峰高 mAU	峰面积 mAU*min	相对峰面积 %	塔板数(EP)	S/N	分离度(EP)
1	7.86	3.159	0.2373	0.16	69247	1.9	39.72
2	13.46	1426.384	144.0231	98.35	109232	874.9	9.35
3	14.79	0.430	0.0343	0.02	228550	0.3	1.92
4	15.04	5.662	0.4817	0.33	192668	3.5	3.01
5	15.50	0.304	0.0356	0.02	137183	0.2	4.59
6	16.19	0.919	0.0766	0.05	238581	0.6	7.08
7	17.22	0.686	0.0683	0.05	184606	0.4	1.21
8	17.40	7.071	0.5919	0.40	273243	4.3	11.49
9	19.18	4.026	0.4638	0.32	184332	2.5	16.77
10	22.25	0.259	0.0316	0.02	225230	0.2	6.65
11	23.28	4.708	0.3934	0.27	563417	2.9	n.a.
总和:		1453.609	146.438	100.00			

Figure S1. HPLC chromatogram of crude linagliptin.

20 杂质20160519

样品名:	**杂质20160519**	*进样量:*	**5.0**
瓶序号:	**RE8**	*通道:*	**UV_VIS_1**
样品类型:	**unknown**	*波长:*	**225.0**
控制程序:	**利格列汀N-008**	*带宽:*	**4**
定量方法:	**利格列汀**	*稀释因子:*	**1.0000**
记录时间:	**2016/5/19 21:05**	*样品重量:*	**1.0000**
运行时间 (min):	**35.00**	*样品量:*	**1.0000**

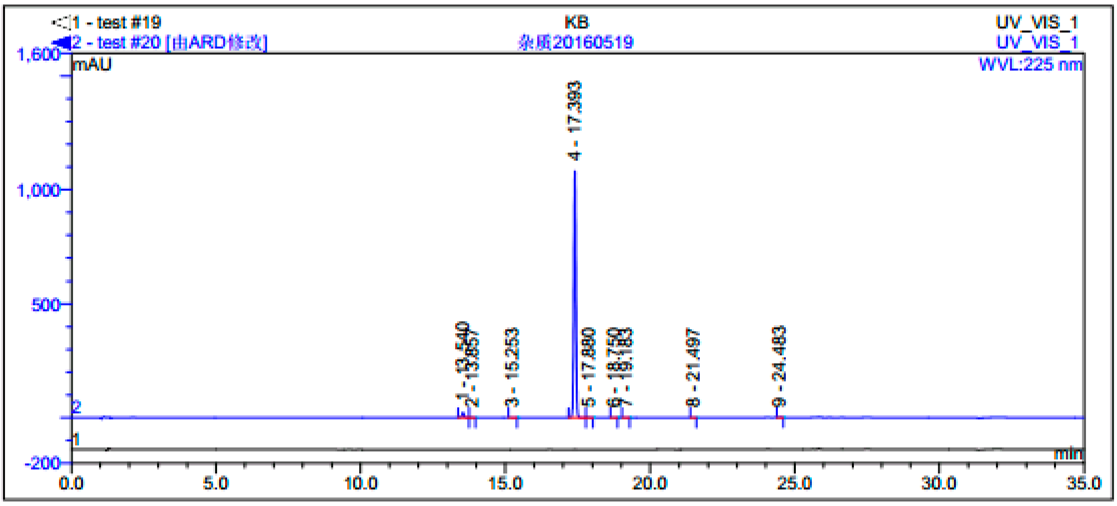

序号	保留时间<br>min	峰高<br>mAU	峰面积<br>mAU*min	相对峰面积<br>%	塔板数(EP)	S/N	分离度(EP)
1	13.54	22.503	1.8188	1.91	180561	841.3	2.59
2	13.86	1.301	0.0963	0.10	220220	48.6	10.00
3	15.25	0.268	0.0288	0.03	141824	10.0	14.40
4	17.39	1082.988	92.6805	97.52	261876	40489.7	3.48
5	17.88	1.048	0.1002	0.11	244177	39.2	4.72
6	18.75	0.166	0.0215	0.02	111218	6.2	2.33
7	19.18	0.879	0.0828	0.09	269352	32.9	14.22
8	21.50	0.191	0.0202	0.02	232206	7.2	19.17
9	24.48	2.292	0.1897	0.20	534356	85.7	n.a.
**总和:**		1111.637	95.039	100.00			

Figure S2. HPLC chromatogram of impurity **2**.

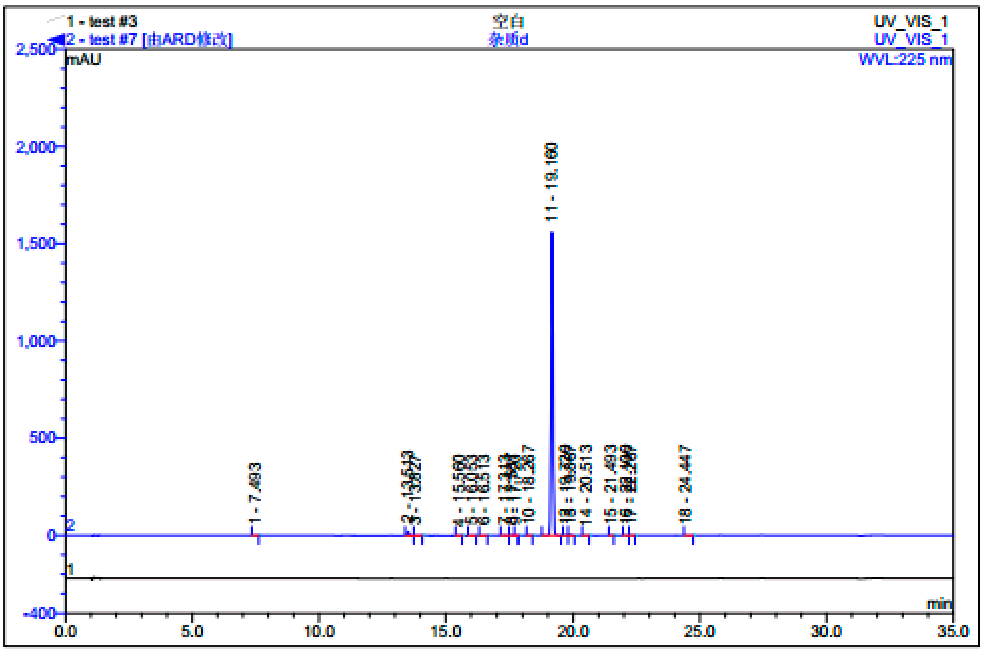

序号	保留时间 min		峰名称	峰高 mAU	峰面积 mAU*min	相对峰面积 %	样品量	类型
1	7.49	n.a.		0.648	0.070	0.05	n.a.	BMB
2	13.51	n.a.		20.123	1.675	1.16	n.a.	BM
3	13.83	n.a.		0.738	0.088	0.06	n.a.	MB
4	15.56	n.a.		0.452	0.045	0.03	n.a.	BMB
5	16.05	n.a.		1.035	0.132	0.09	n.a.	BMB
6	16.51	n.a.		0.577	0.073	0.05	n.a.	BMB
7	17.31	n.a.		1.342	0.205	0.14	n.a.	BM
8	17.59	n.a.		2.589	0.270	0.19	n.a.	MB
9	17.72	n.a.		0.332	0.025	0.02	n.a.	Rd
10	18.27	n.a.		0.956	0.082	0.06	n.a.	BMB
11	19.16	n.a.		1559.974	140.387	97.34	n.a.	BMB
12	19.72	n.a.		1.863	0.190	0.13	n.a.	BM
13	19.89	n.a.		4.980	0.549	0.38	n.a.	MB
14	20.51	n.a.		0.334	0.039	0.03	n.a.	BMB
15	21.49	n.a.		1.561	0.118	0.08	n.a.	BMB

Figure S3. HPLC chromatogram of impurity **3**.

14 杂质20160517

样品名:	**杂质20160517**	*进样量:*	**5.0**
瓶序号:	**RE3**	*通道:*	**UV_VIS_1**
样品类型:	**unknown**	*波长:*	**225.0**
控制程序:	**利格列汀N-008**	*带宽:*	**4**
定量方法:	**利格列汀**	*稀释因子:*	**1.0000**
记录时间:	**2016/5/19 17:29**	*样品重量:*	**1.0000**
运行时间 (min):	**35.00**	*样品量:*	**1.0000**

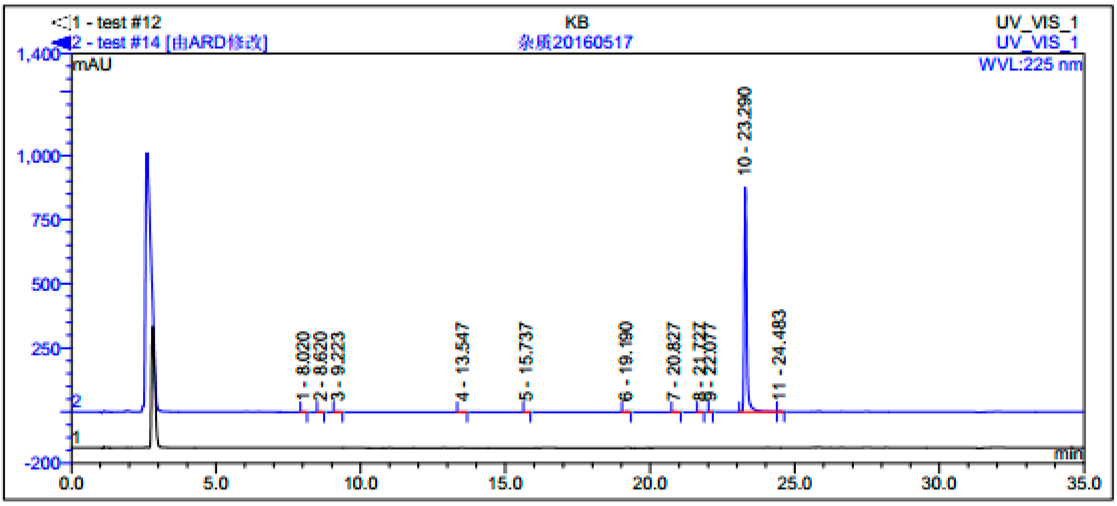

序号	保留时间 min	峰高 mAU	峰面积 mAU*min	相对峰面积 %	塔板数(EP)	S/N	分离度(EP)
1	8.02	0.224	0.0244	0.03	29008	2.2	3.34
2	8.62	0.258	0.0274	0.03	40487	2.6	3.50
3	9.22	0.719	0.0817	0.10	45004	7.2	28.45
4	13.55	0.944	0.0828	0.10	171472	9.5	16.22
5	15.74	0.249	0.0244	0.03	202388	2.5	23.97
6	19.19	2.006	0.1891	0.23	265455	20.2	11.34
7	20.83	0.508	0.0522	0.06	351632	5.1	5.35
8	21.73	0.285	0.0337	0.04	194348	2.9	2.29
9	22.08	0.385	0.0258	0.03	645678	3.9	9.97
10	23.29	876.298	83.0714	99.15	481498	8806.7	8.87
11	24.48	2.016	0.1708	0.20	521054	20.3	n.a.
总和:		883.891	83.784	100.00			

Figure S4. HPLC chromatogram of impurity **4**.

19 杂质-20160419

样品名:	**杂质-20160419**	*进样量:*	**8.0**
瓶序号:	**BC2**	*通道:*	**UV_VIS_1**
样品类型:	**unknown**	*波长:*	**225.0**
控制程序:	**利格列汀N-008**	*带宽:*	**4**
定量方法:	**利格列汀**	*稀释因子:*	**1.0000**
记录时间:	**2016/4/26 2:38**	*样品重量:*	**1.0000**
运行时间 (min):	**35.00**	*样品量:*	**1.0000**

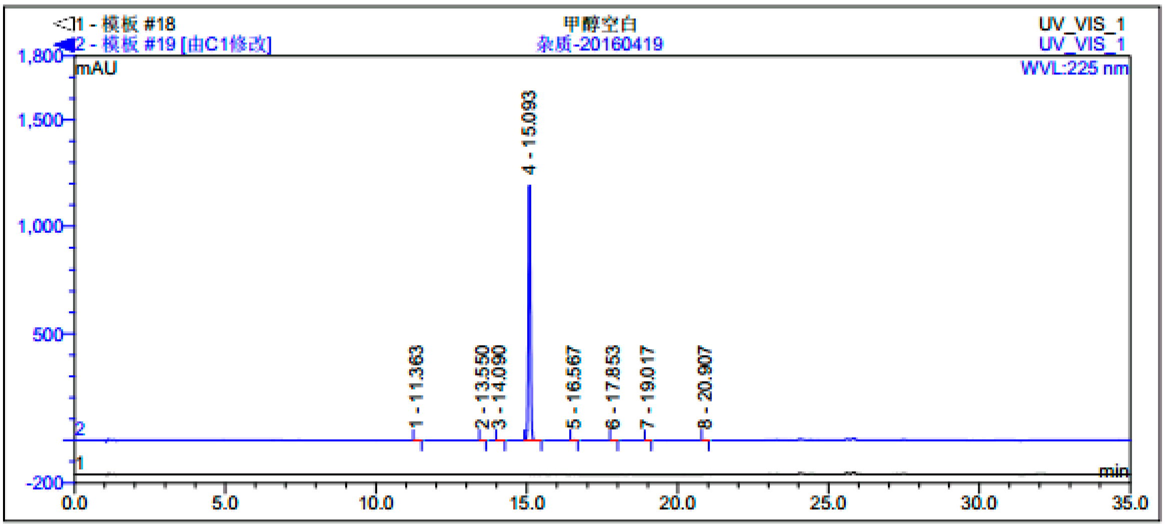

序号	保留时间 min	峰高 mAU	峰面积 mAU*min	相对峰面积 %	塔板数(EP)	S/N	分离度(EP)
1	11.36	1.066	0.0929	0.09	110848	15.4	16.19
2	13.55	0.509	0.0422	0.04	162980	7.4	3.85
3	14.09	0.409	0.0411	0.04	147562	5.9	7.06
4	15.09	1192.397	103.7794	99.61	190784	17273.6	10.81
5	16.57	0.770	0.0660	0.06	240572	11.2	8.83
6	17.85	0.226	0.0234	0.02	206379	3.3	7.73
7	19.02	0.247	0.0220	0.02	276210	3.6	13.02
8	20.91	1.301	0.1184	0.11	326138	18.8	n.a.
总和:		1196.924	104.185	100.00			

Figure S5. HPLC chromatogram of impurity **5**.

样品名称:	杂质e	进样体积:	3.0
样品瓶号:	BB4	通道:	UV_VIS_1
样品类型:	unknown	波长:	225
控制程序:	LGLTN008	带宽:	n. a.
定量方法:	test	稀释因子:	1.0000
记录时间:	6-2-16 20:25	样品重量:	1.0000
运行时间:	26.90	内标量:	1.0000

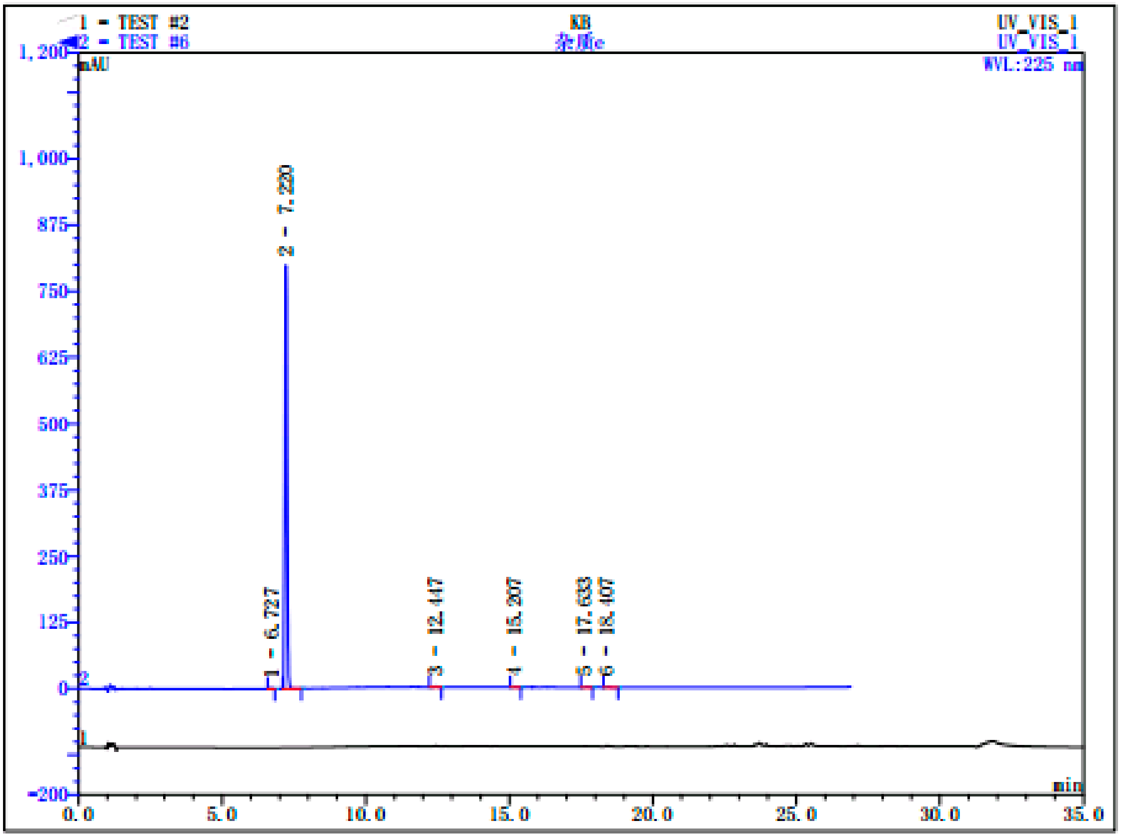

序号	峰名称	保留时间 (检测) min	峰面积 mAU*min	相对峰面积 %	峰高 mAU	样品量
1	n.a.	6.73	0.135	0.19	1.694	n.a.
2	n.a.	7.22	68.845	98.76	798.382	n.a.
3	n.a.	12.45	0.213	0.31	1.841	n.a.
4	n.a.	15.21	0.093	0.13	1.008	n.a.
5	n.a.	17.63	0.174	0.25	1.068	n.a.
6	n.a.	18.41	0.248	0.36	1.998	n.a.

Figure S6. HPLC chromatogram of impurity **6**.

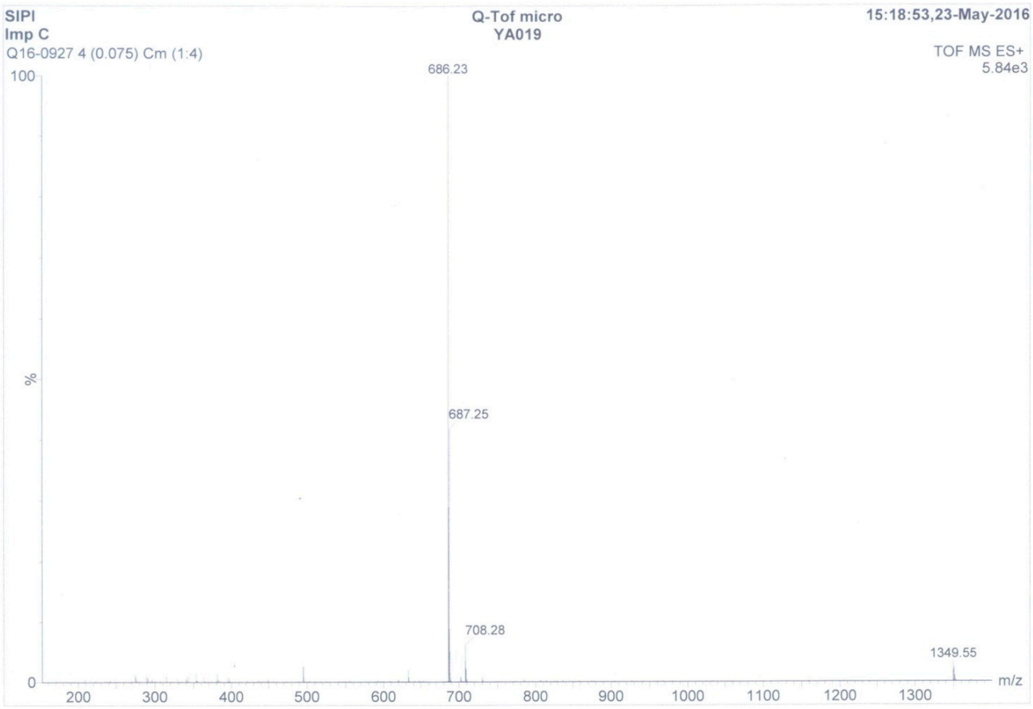

Figure S7. MS spectrogram of impurity **2**.

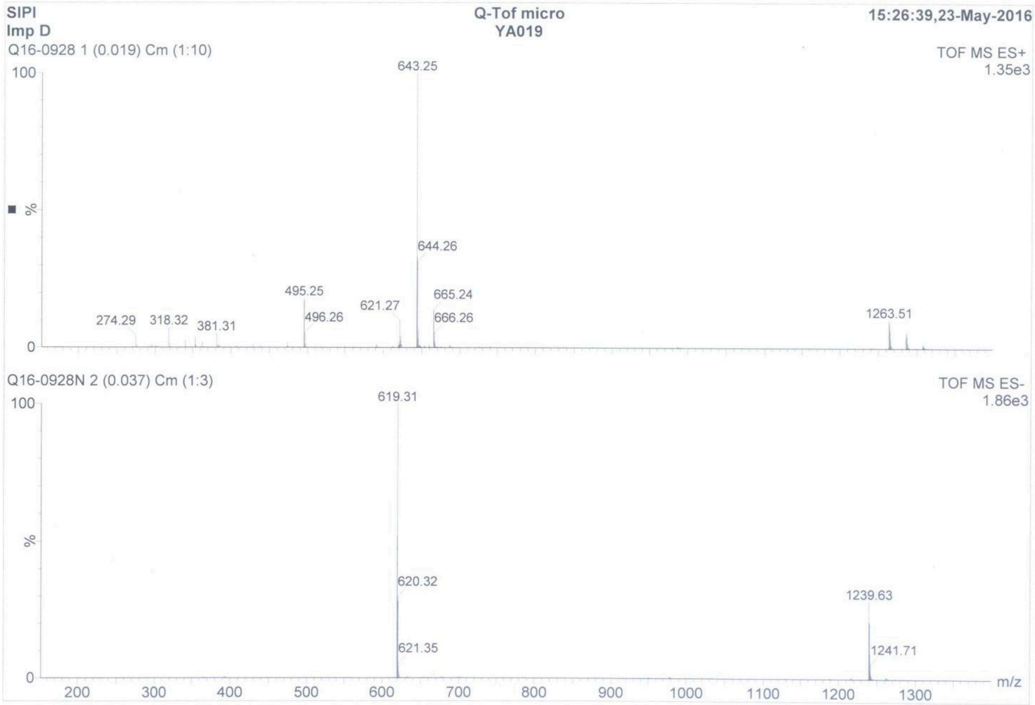

Figure S8. MS spectrogram of impurity **3**.

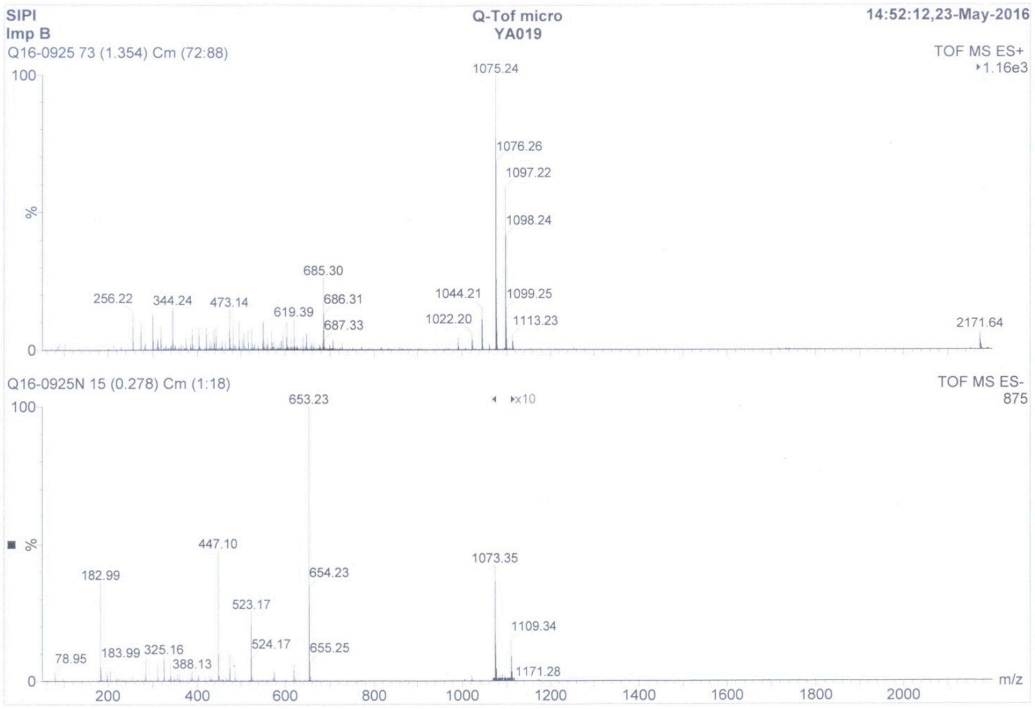

Figure S9. MS spectrogram of impurity **4**.

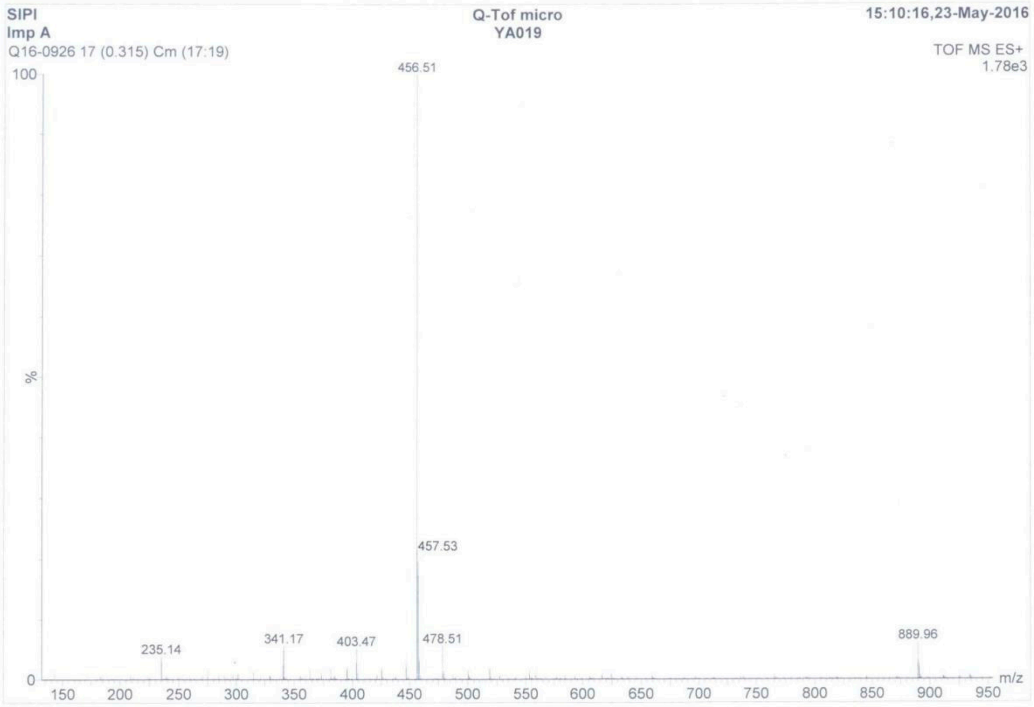

Figure S10. MS spectrogram of impurity **5**.

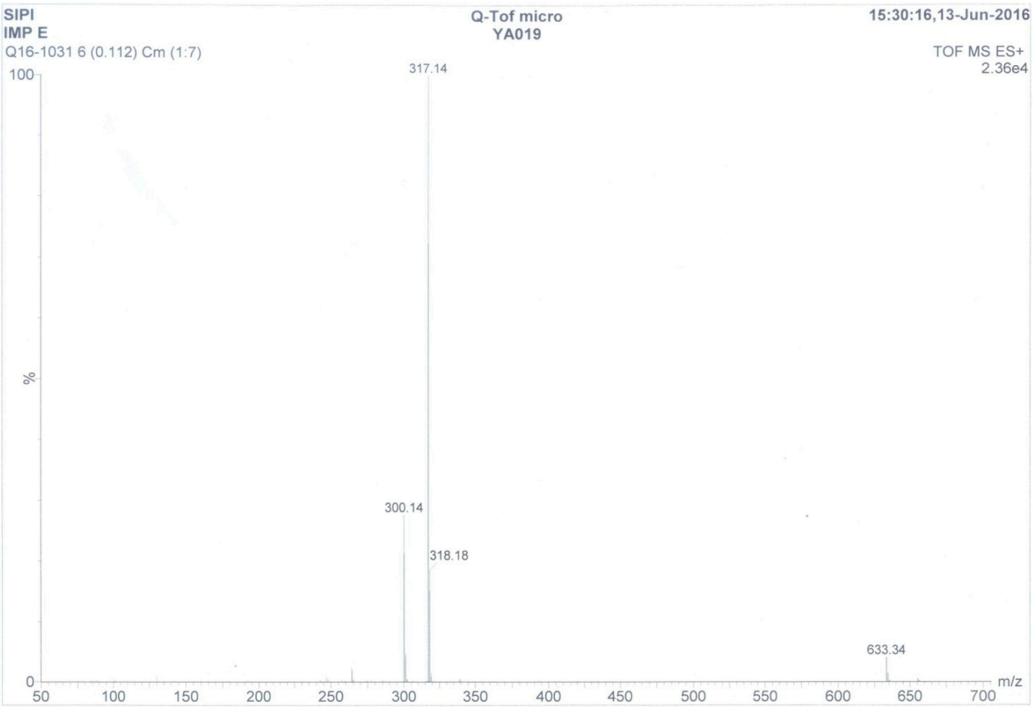

Figure S11. MS spectrogram of impurity **6**.

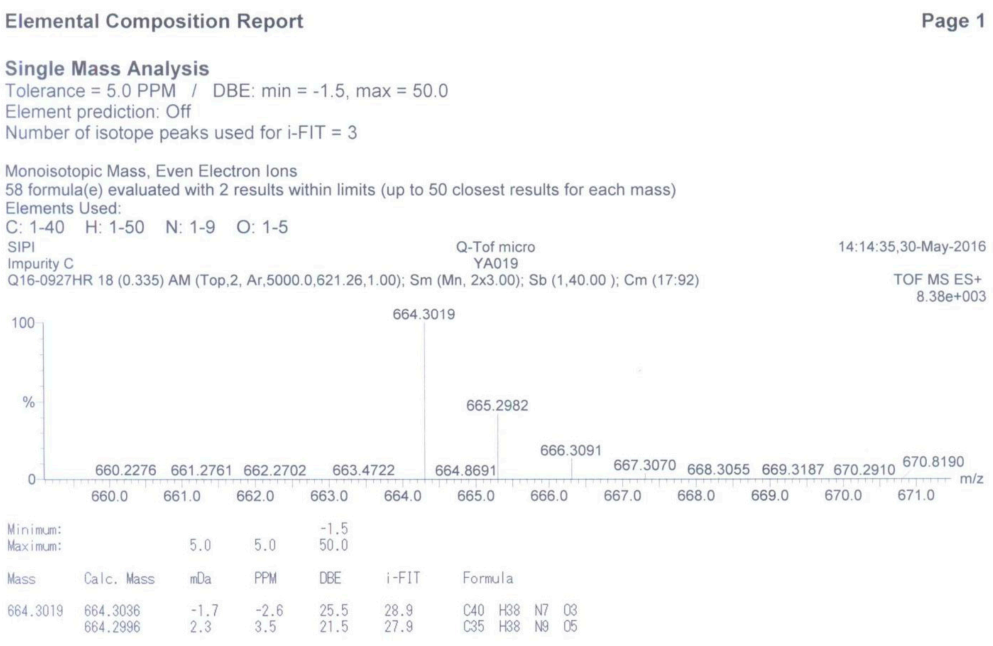
Elemental Composition Report

Page 1

Single Mass Analysis
Tolerance = 5.0 PPM / DBE: min = -1.5, max = 50.0
Element prediction: Off
Number of isotope peaks used for i-FIT = 3

Monoisotopic Mass, Even Electron Ions
58 formula(e) evaluated with 2 results within limits (up to 50 closest results for each mass)
Elements Used:
C: 1-40 H: 1-50 N: 1-9 O: 1-5

Minimum:				-1.5		
Maximum:		5.0	5.0	50.0		
Mass	Calc. Mass	mDa	PPM	DBE	i-FIT	Formula
664.3019	664.3036	-1.7	-2.6	25.5	28.9	C40 H38 N7 O3
	664.2996	2.3	3.5	21.5	27.9	C35 H38 N9 O5

Figure S12. HRMS spectrogram of impurity **2**.

Elemental Composition Report Page 1

Single Mass Analysis
Tolerance = 5.0 PPM / DBE: min = -1.5, max = 50.0
Element prediction: Off
Number of isotope peaks used for i-FIT = 3

Monoisotopic Mass, Even Electron Ions
89 formula(e) evaluated with 2 results within limits (up to 50 closest results for each mass)
Elements Used:
C: 1-40 H: 1-50 N: 1-8 O: 1-5
SIPI Q-Tof micro 13:58:25,30-May-2016
Impurity D YA019
Q16-0928H 15 (0.280) AM (Cen,2, 80.00, Ar,5000.0,664.30,1.00); Sm (Mn, 2x3.00); Sb (1,40.00); Cm (7:31) TOF MS ES+ 4.72e+002

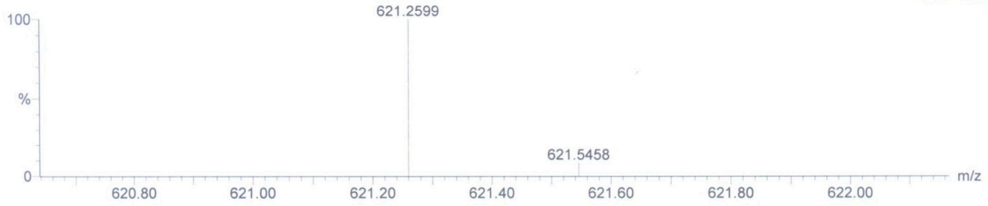

Mass	Calc. Mass	mDa	PPM	DBE	i-FIT	Formula
Minimum:				-1.5		
Maximum:		5.0	5.0	50.0		
621.2599	621.2614	-1.5	-2.4	25.5	n/a	C38 H33 N6 O3
	621.2574	2.5	4.0	21.5	n/a	C33 H33 N8 O5

Figure S13. HRMS spectrogram of impurity **3**.

Elemental Composition Report Page 1

Single Mass Analysis
Tolerance = 5.0 PPM / DBE: min = -1.5, max = 50.0
Element prediction: Off
Number of isotope peaks used for i-FIT = 3

Monoisotopic Mass, Even Electron Ions
116 formula(e) evaluated with 2 results within limits (up to 50 closest results for each mass)
Elements Used:
C: 1-65 H: 1-65 N: 1-16 O: 1-6
SIPI Q-Tof micro 16:02:51,30-May-2016
Impurity B YA019
Q16-0925HR 4 (0.075) AM (Top,2, Ar,5000.0,1069.43,1.00); Sm (Mn, 2x3.00); Sb (1,40.00); Cm (4:22) TOF MS ES+ 2.26e+001

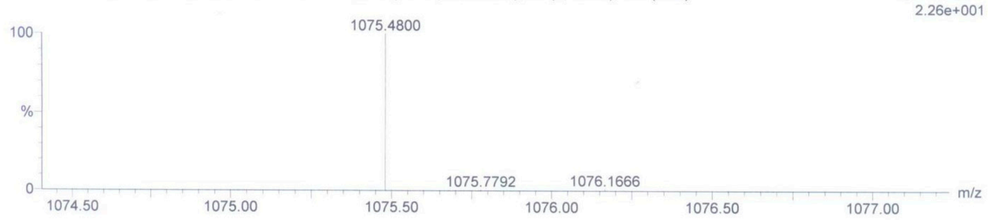

Mass	Calc. Mass	mDa	PPM	DBE	i-FIT	Formula
Minimum:				-1.5		
Maximum:		5.0	5.0	50.0		
1075.4800	1075.4803	-0.3	-0.3	37.5	n/a	C58 H59 N16 O6
	1075.4844	-4.4	-4.1	41.5	n/a	C63 H59 N14 O4

Figure S14. HRMS spectrogram of impurity **4**.

Elemental Composition Report Page 1

Single Mass Analysis
Tolerance = 5.0 PPM / DBE: min = -1.5, max = 50.0
Element prediction: Off
Number of isotope peaks used for i-FIT = 3

Monoisotopic Mass, Even Electron Ions
94 formula(e) evaluated with 1 results within limits (up to 50 closest results for each mass)
Elements Used:
C: 1-40 H: 1-55 N: 1-7 O: 1-3
SIPI Q-Tof micro 15:32:17,30-May-2016
Impurity A YA019
Q16-0926HR 55 (1.021) AM (Top,2, Ar,5000.0,432.14,1.00); Sm (Mn, 2x3.00); Sb (1,40.00); Cm (22:60) TOF MS ES+ 1.94e+004

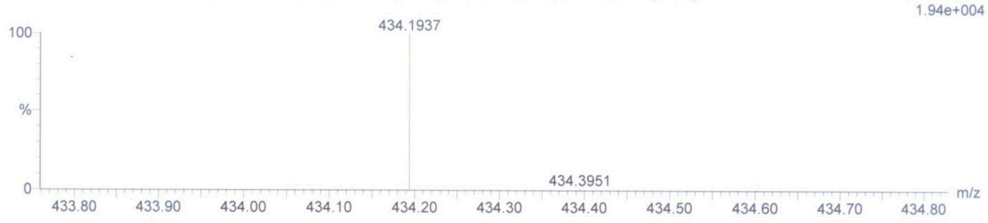

Minimum:				-1.5		
Maximum:		5.0	5.0	50.0		
Mass	Calc. Mass	mDa	PPM	DBE	i-FIT	Formula
434.1937	434.1941	-0.4	-0.9	14.5	n/a	C22 H24 N7 O3

Figure S15. HRMS spectrogram of impurity **5**.

Qualitative Compound Report

**Data File**	20160620-002.d	**Sample Name**	impurity E
**Sample Type**	Sample	**Position**	Vial 71
**Instrument Name**	Instrument 1	**User Name**	
**Acq Method**		**IRM Calibration Status**	Success
**DA Method**	MS.m	**Comment**	N20160620017

Compound Table

Compound Label	RT	Mass	Abund	Formula	Tgt Mass	Diff (ppm)
Cpd 1: C15 H20 N6 O2	0.31	316.1641	135441	C15 H20 N6 O2	316.1648	-2.07

Compound Label	RT	Algorithm	Mass
Cpd 1: C15 H20 N6 O2	0.31	Find By Formula	316.1641

MS Zoomed Spectrum

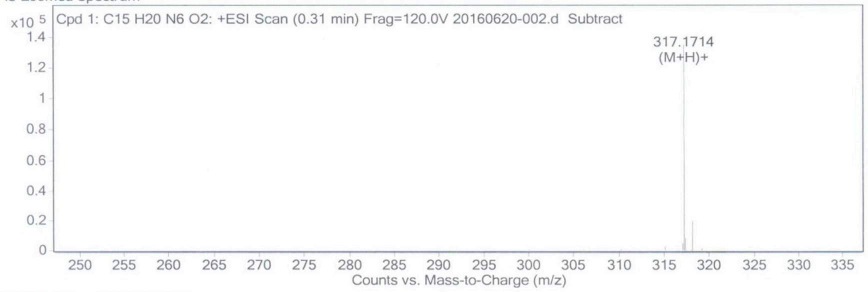

MS Spectrum Peak List

m/z	*Calc m/z*	Diff(ppm)	Abund	Formula	Ion
317.1714	317.1721	-2.06	135441	C15 H21 N6 O2	(M+H)+

--- End Of Report ---

Figure S16. HRMS spectrogram of impurity **6**.

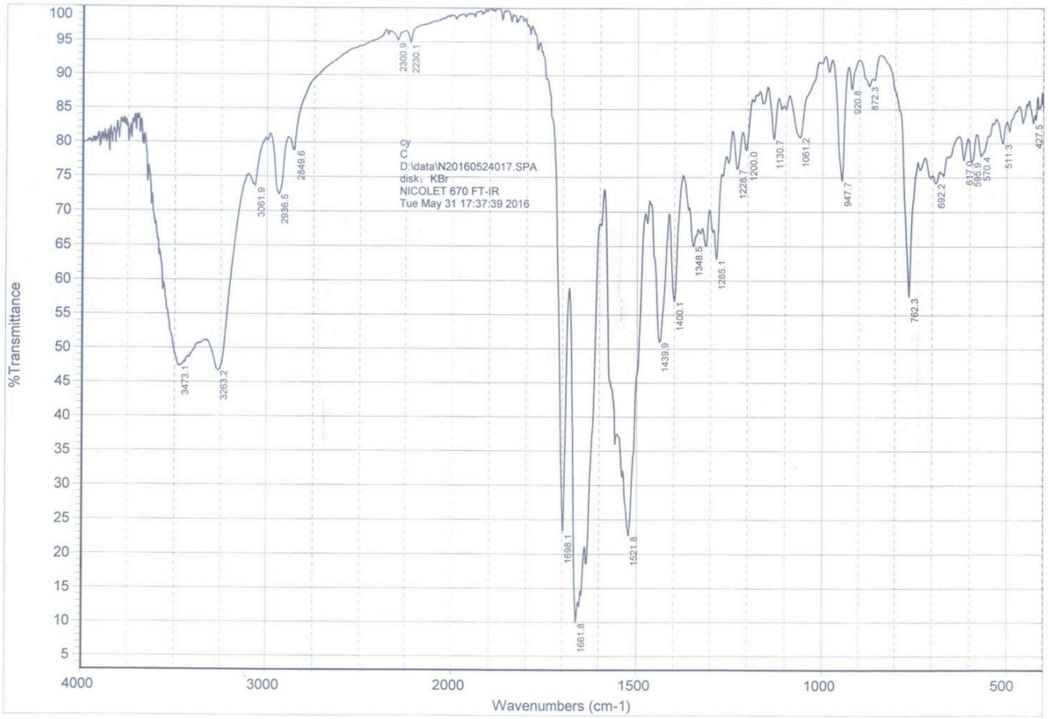

Figure S17. IR spectrogram of impurity **2**.

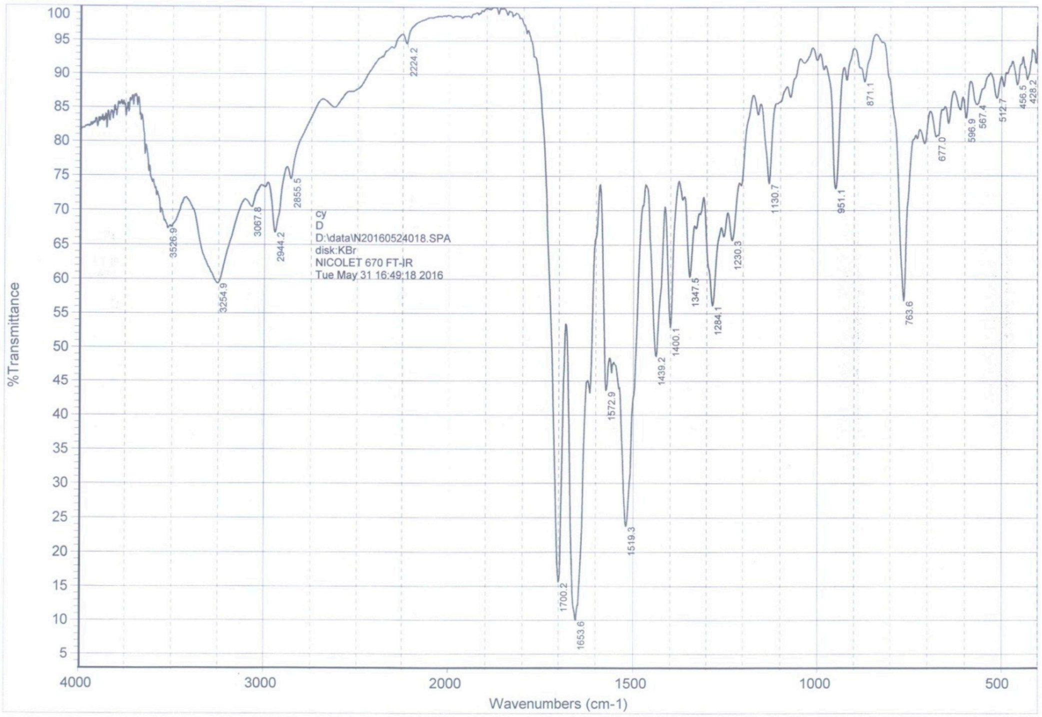

Figure S18. IR spectrogram of impurity **3**.

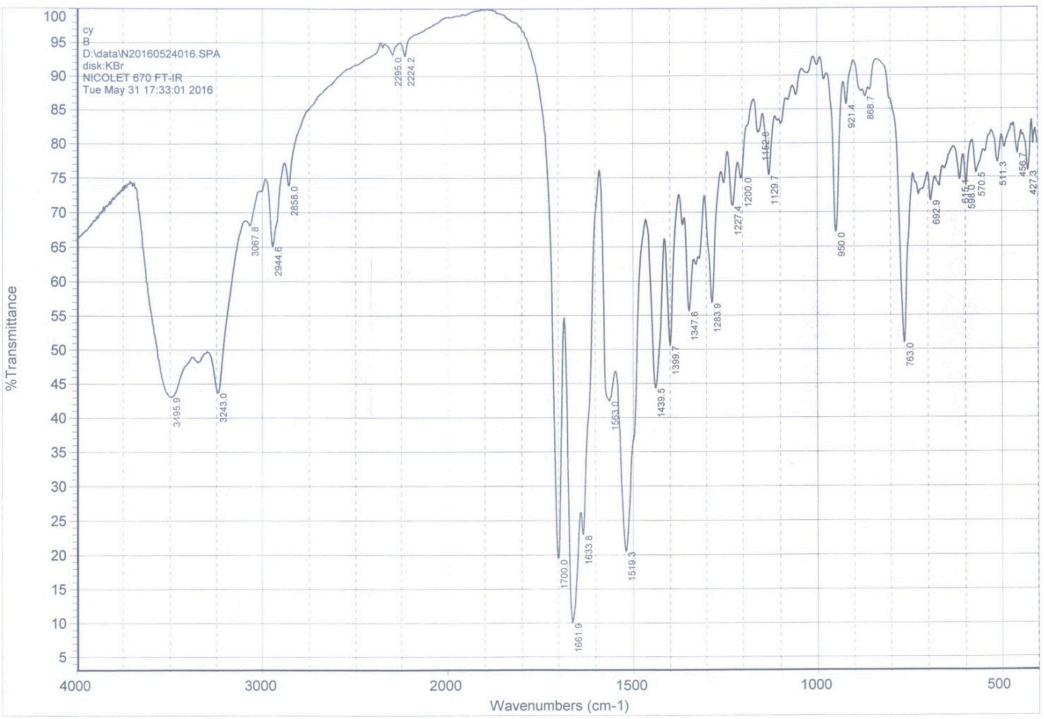

Figure S19. IR spectrogram of impurity **4**.

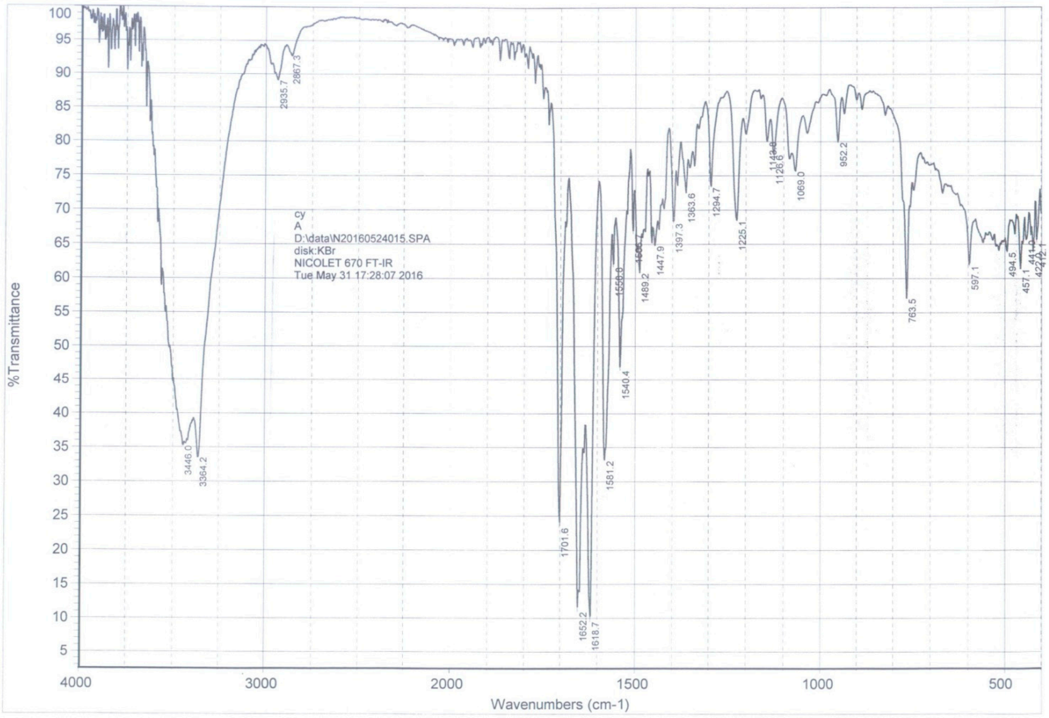

Figure S20. IR spectrogram of impurity **5**.

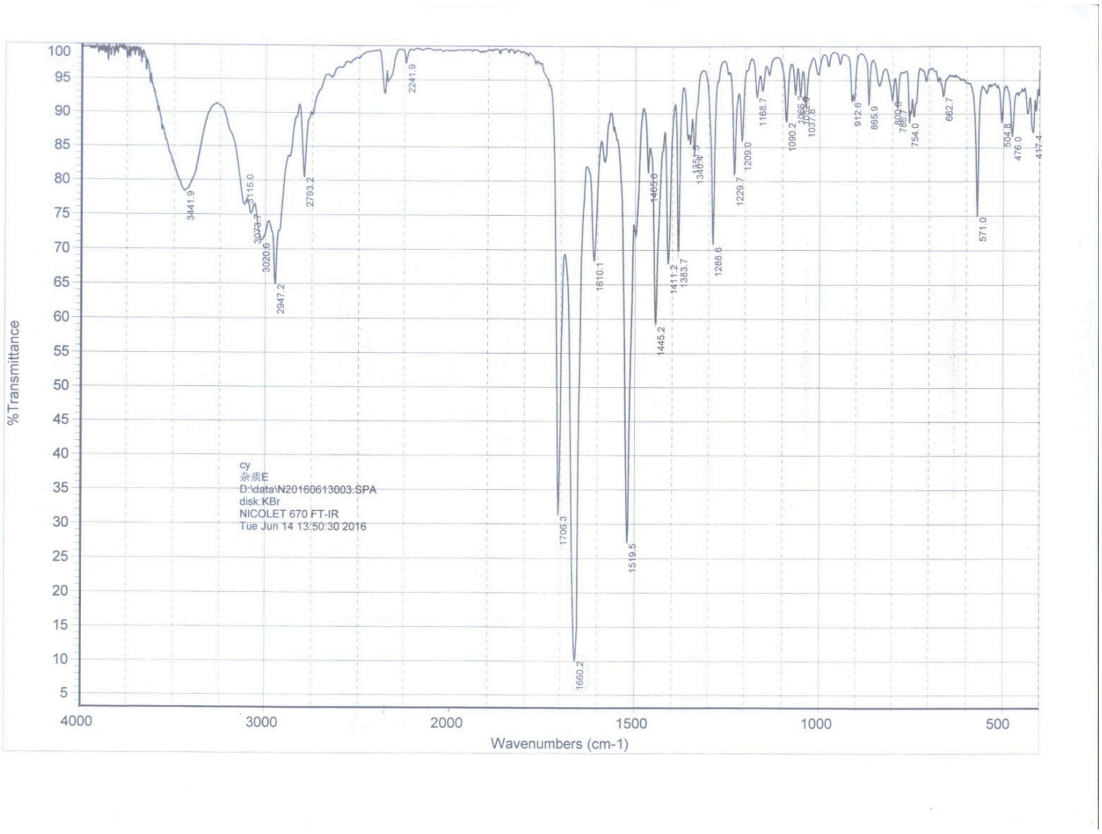

Figure S21. IR spectrogram of impurity **6**.

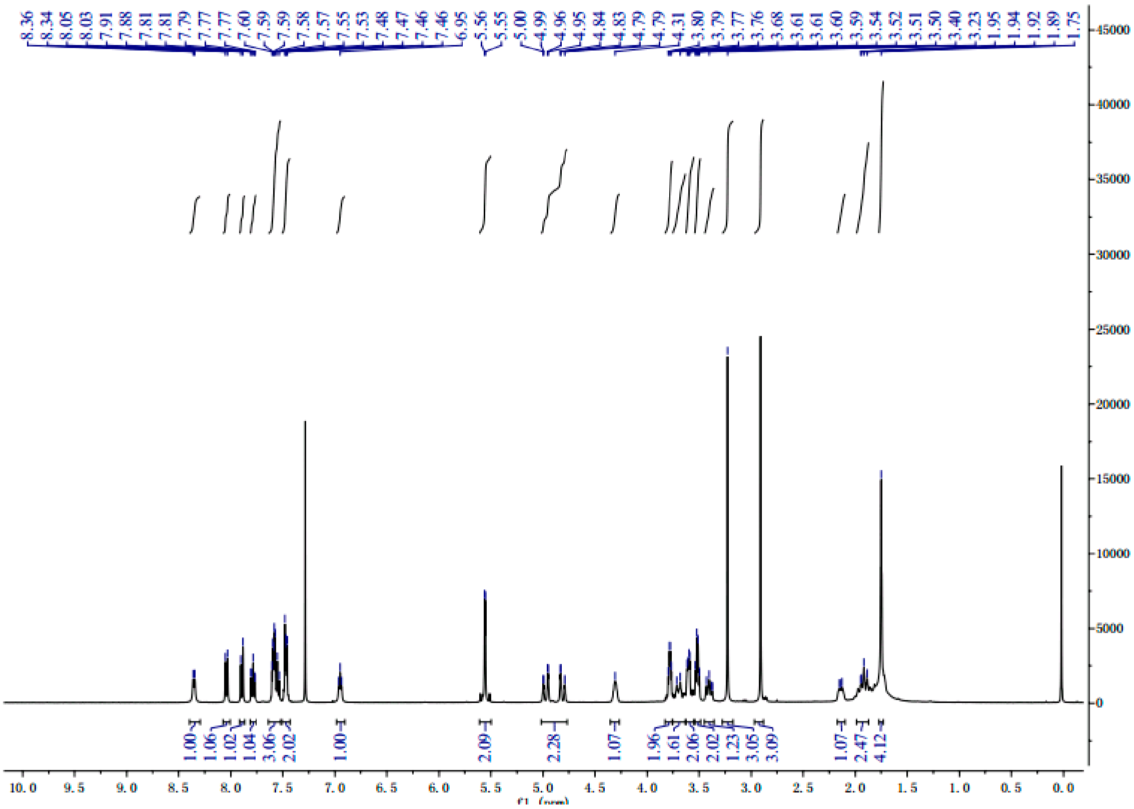

Figure S22. ^{1}H-NMR spectrogram of impurity **2**.

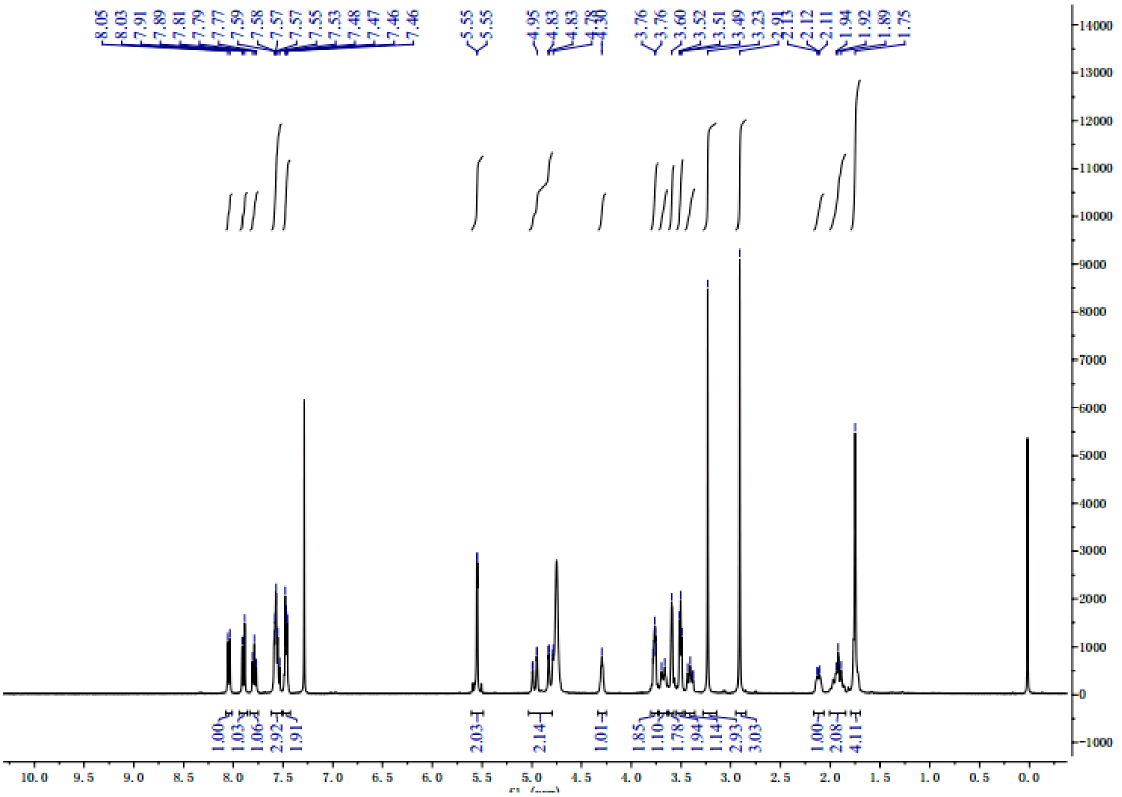

Figure S23. ^{1}H-NMR spectrogram with D_2O added of impurity **2**.

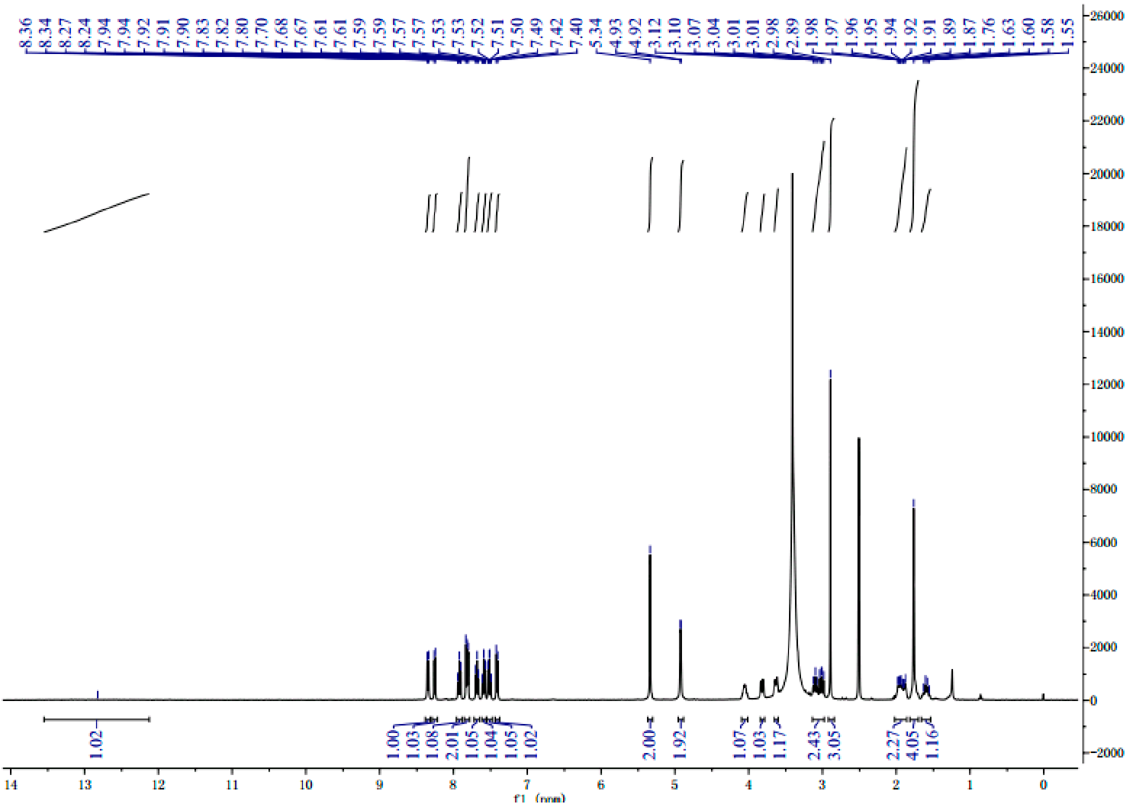

Figure S24. ^{1}H-NMR spectrogram of impurity **3**.

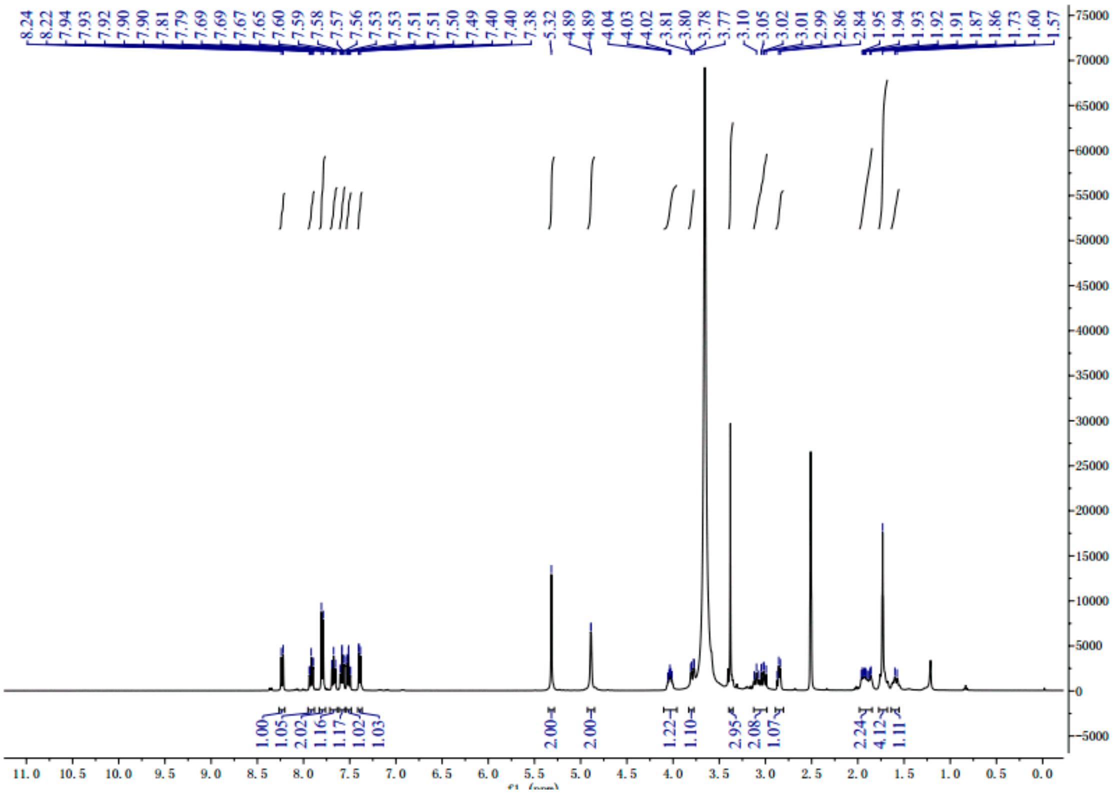

Figure S25. ^{1}H-NMR spectrogram with D_2O added of impurity **3**.

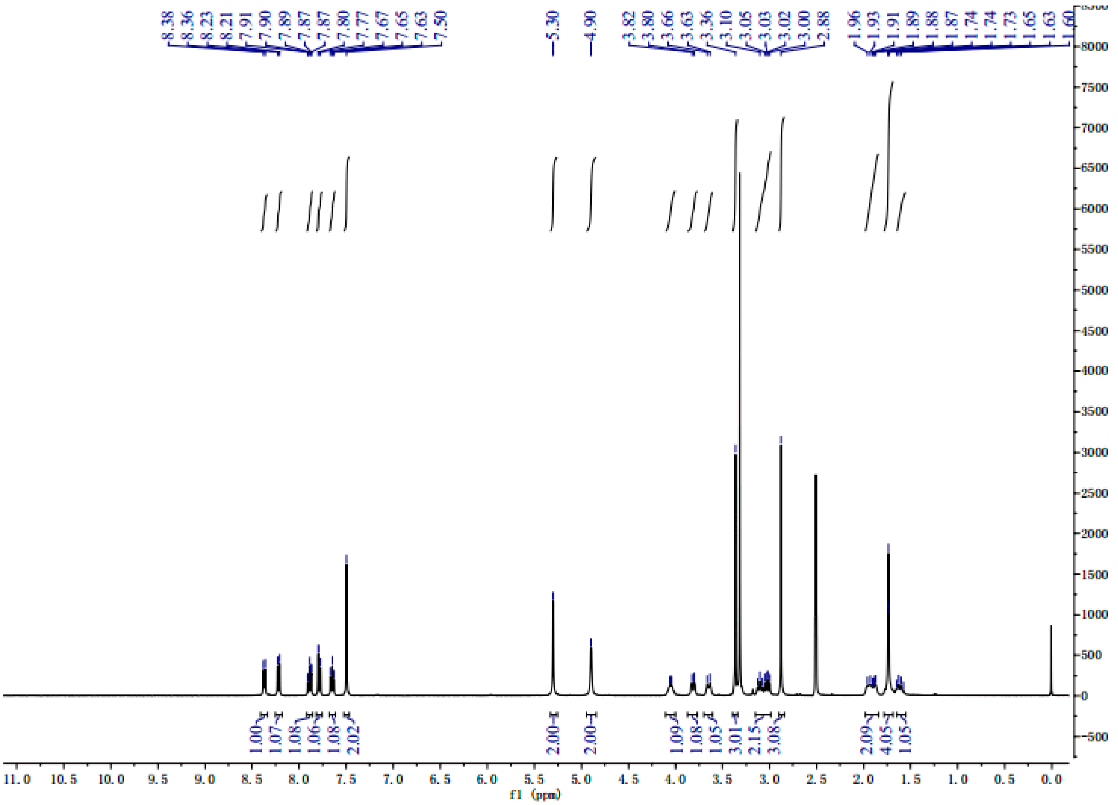

Figure S26. ^{1}H-NMR spectrogram of impurity **4**.

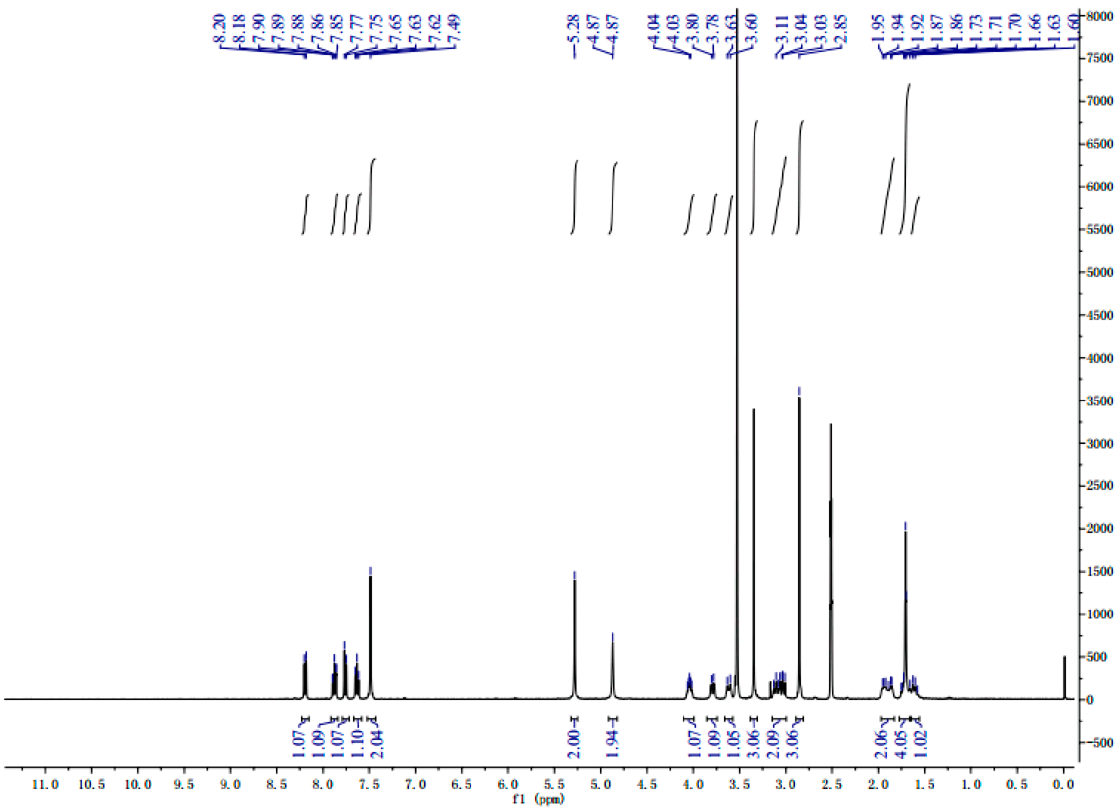

Figure S27. ^{1}H-NMR spectrogram with D_2O added of impurity **4**.

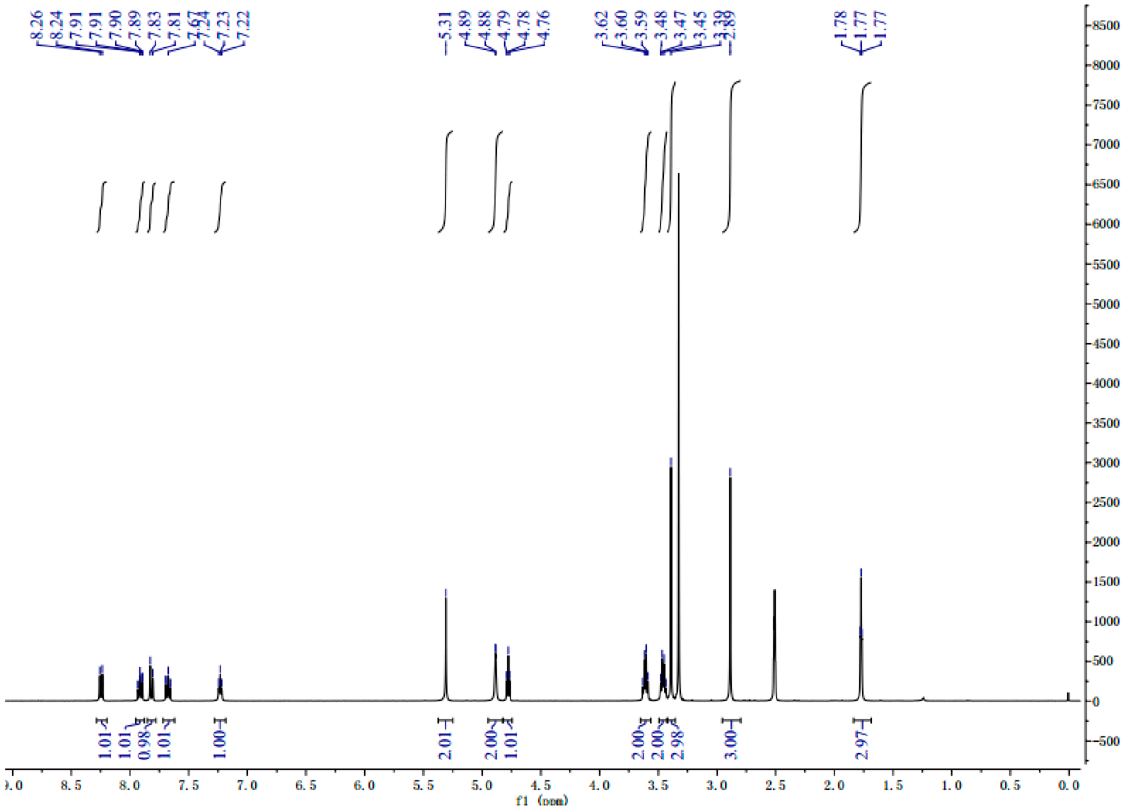

Figure S28. ^{1}H-NMR spectrogram of impurity **5**.

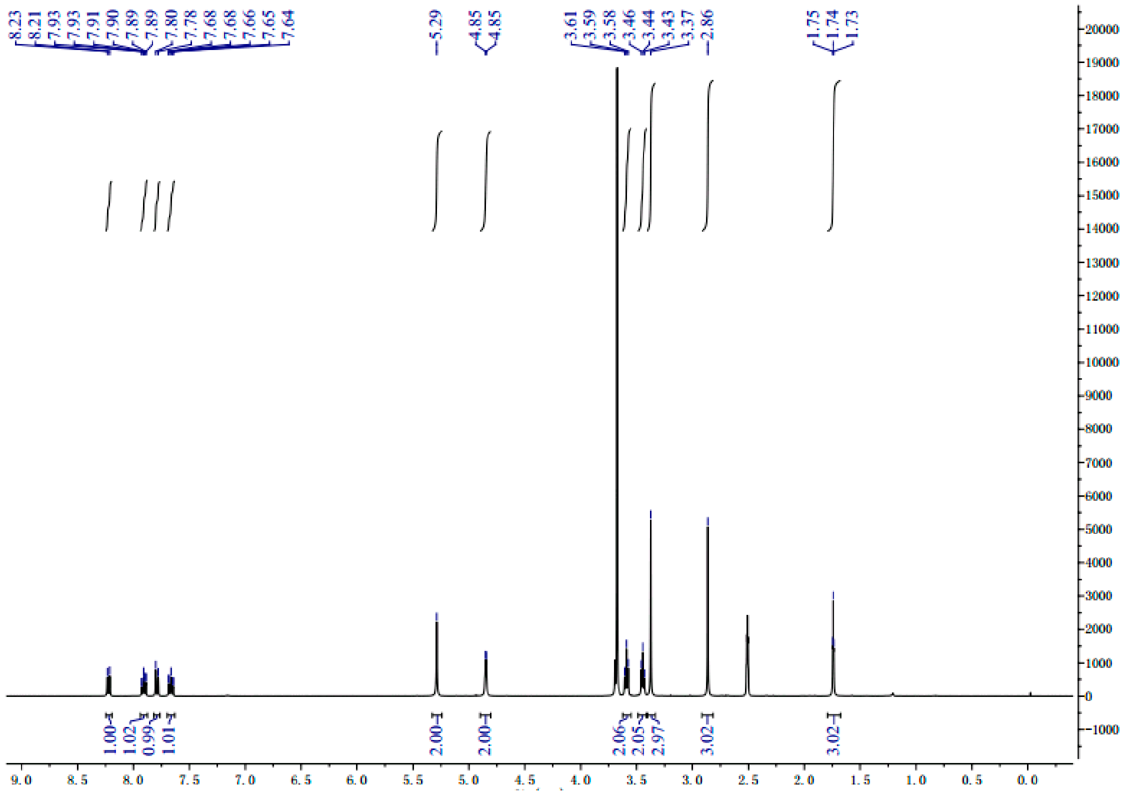

Figure S29. ^{1}H-NMR spectrogram with D_2O added of impurity **5**.

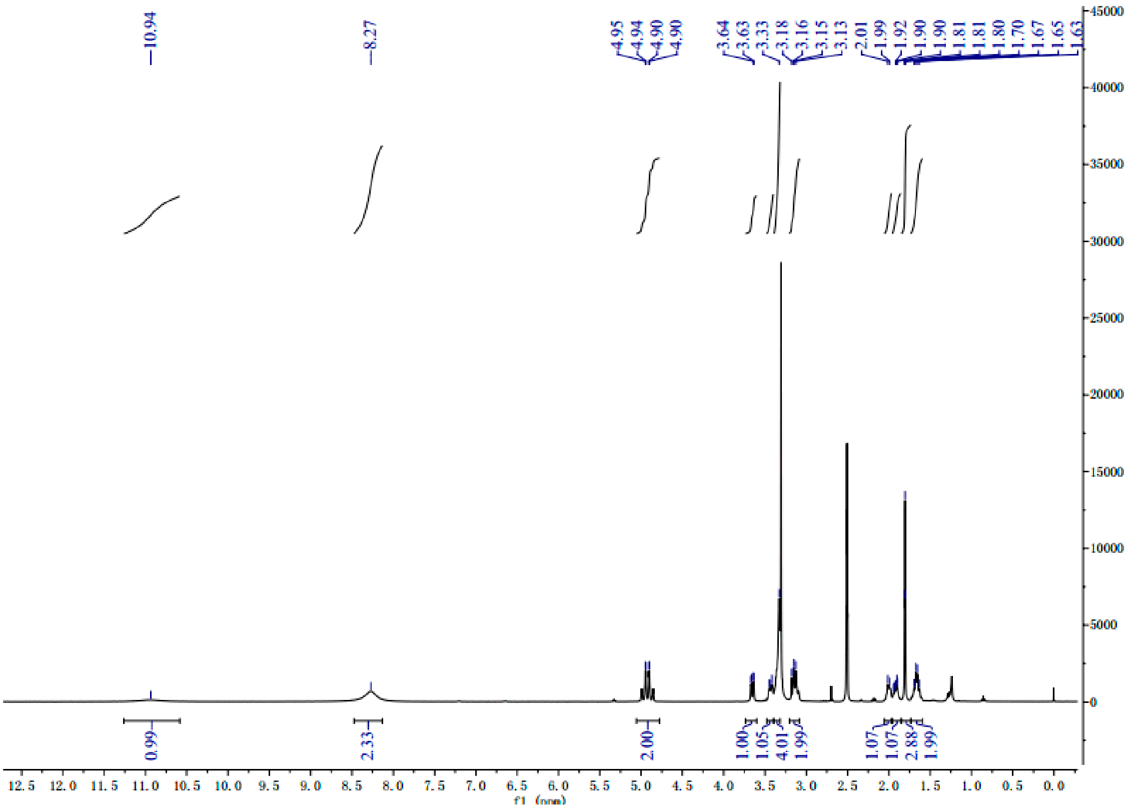

Figure S30. ^{1}H-NMR spectrogram of impurity **6**.

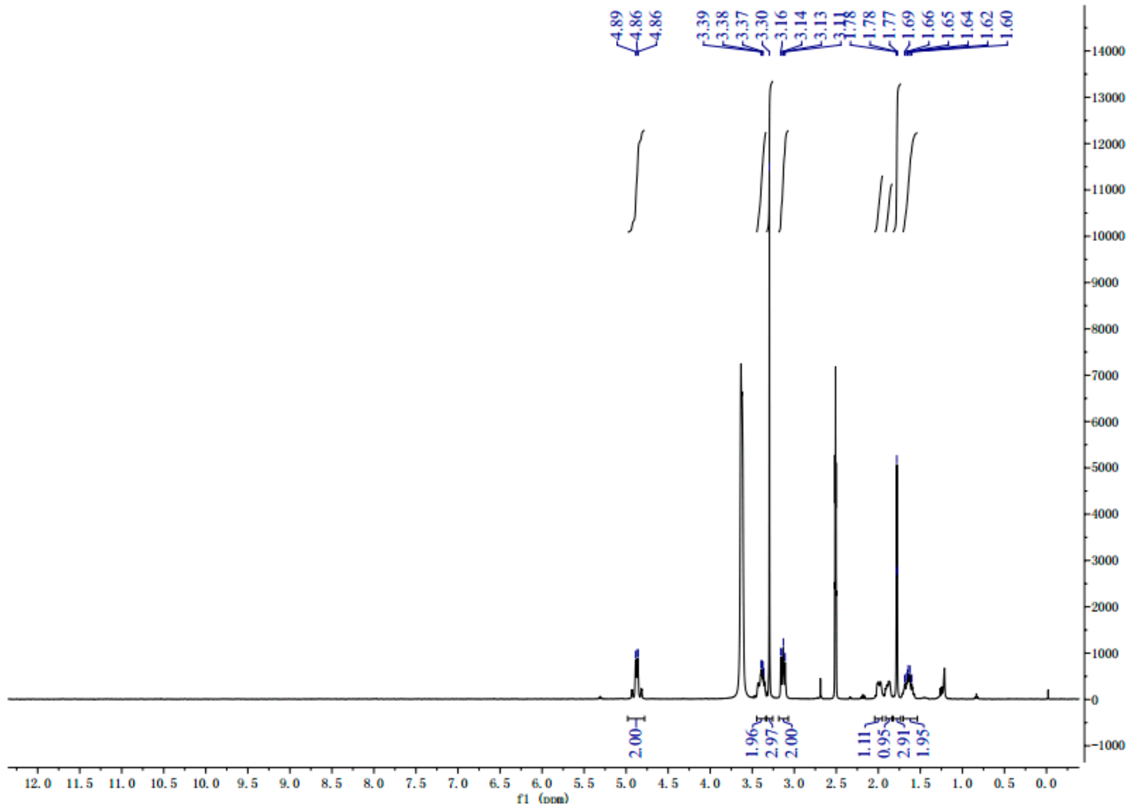

Figure S31. ^{1}H-NMR spectrogram with D_2O added of impurity **6**.

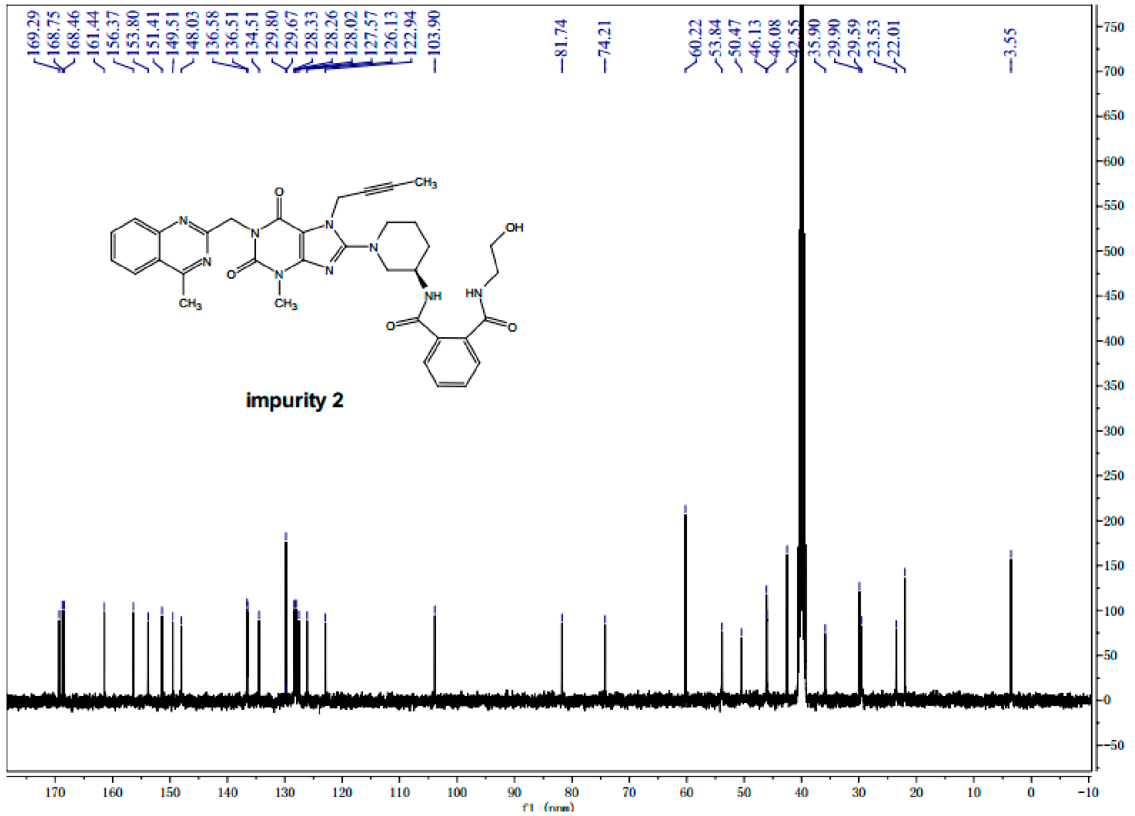

Figure S32. ^{13}C-NMR spectrogram of impurity **2**.

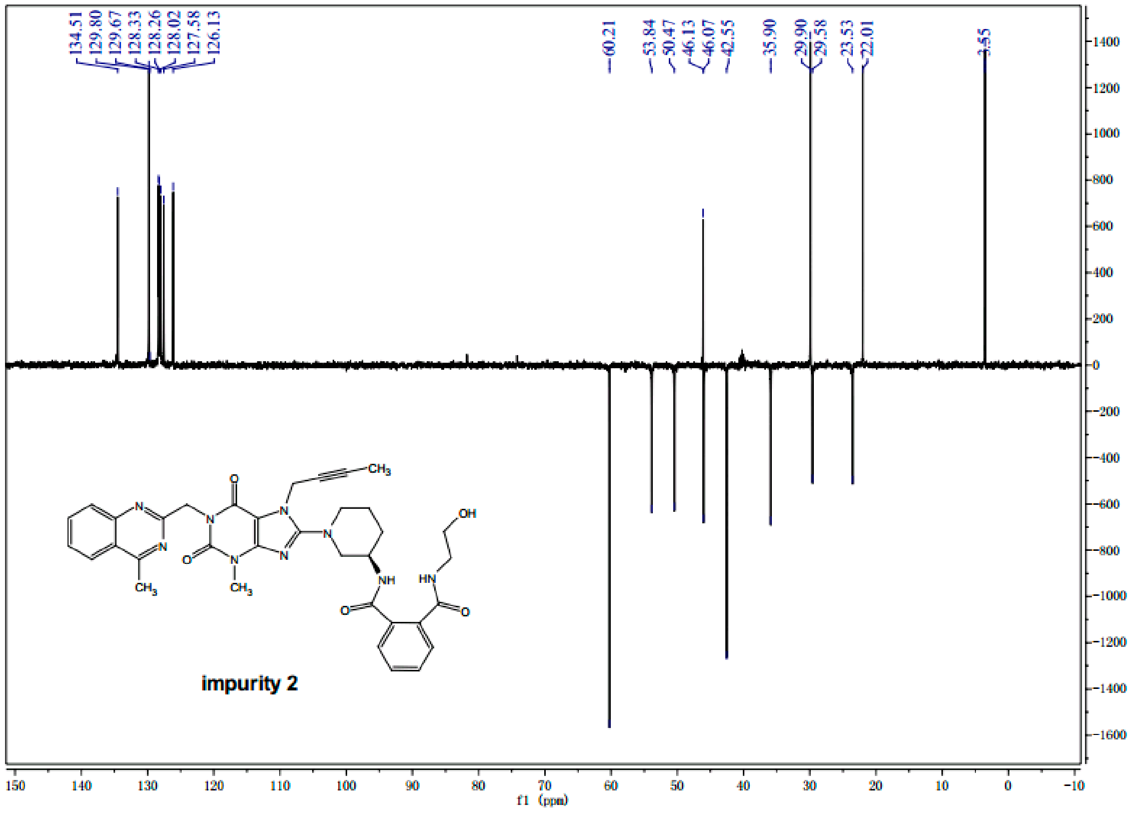

Figure S33. DEPT of impurity **2**.

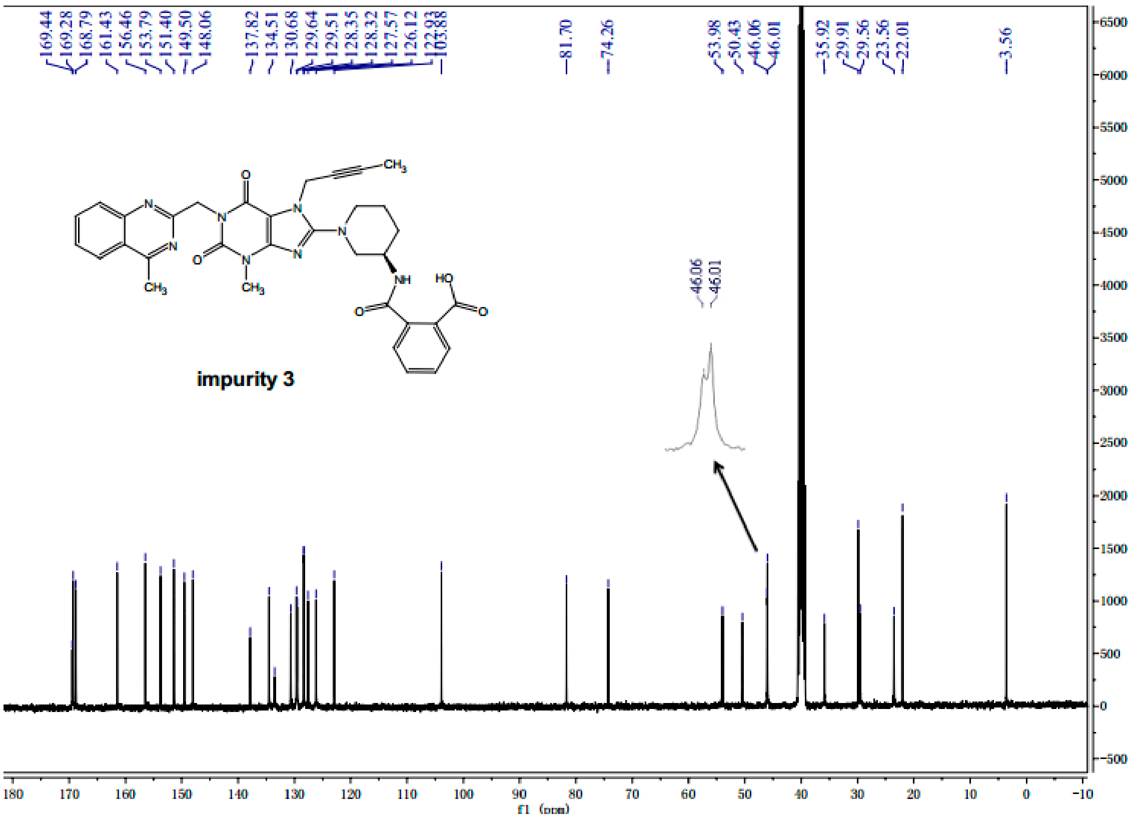

Figure S34. ^{13}C-NMR spectrogram of impurity **3**.

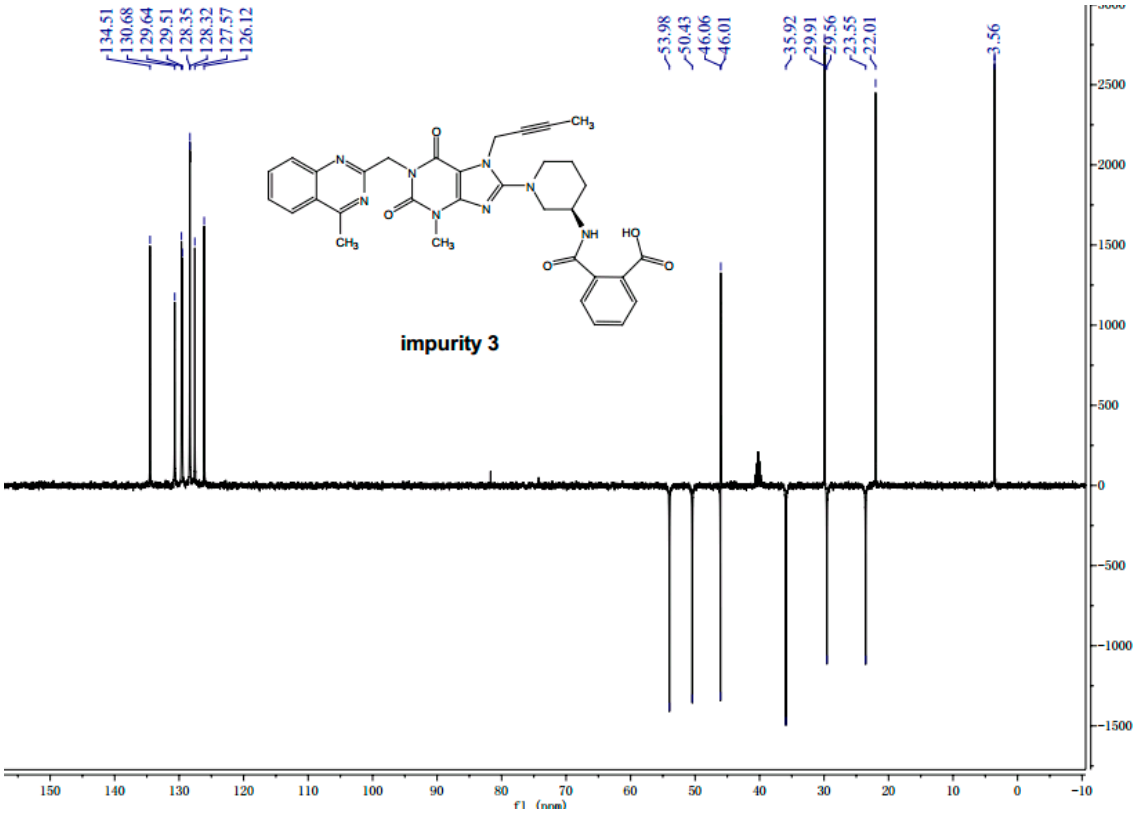

Figure S35. DEPT of impurity **3**.

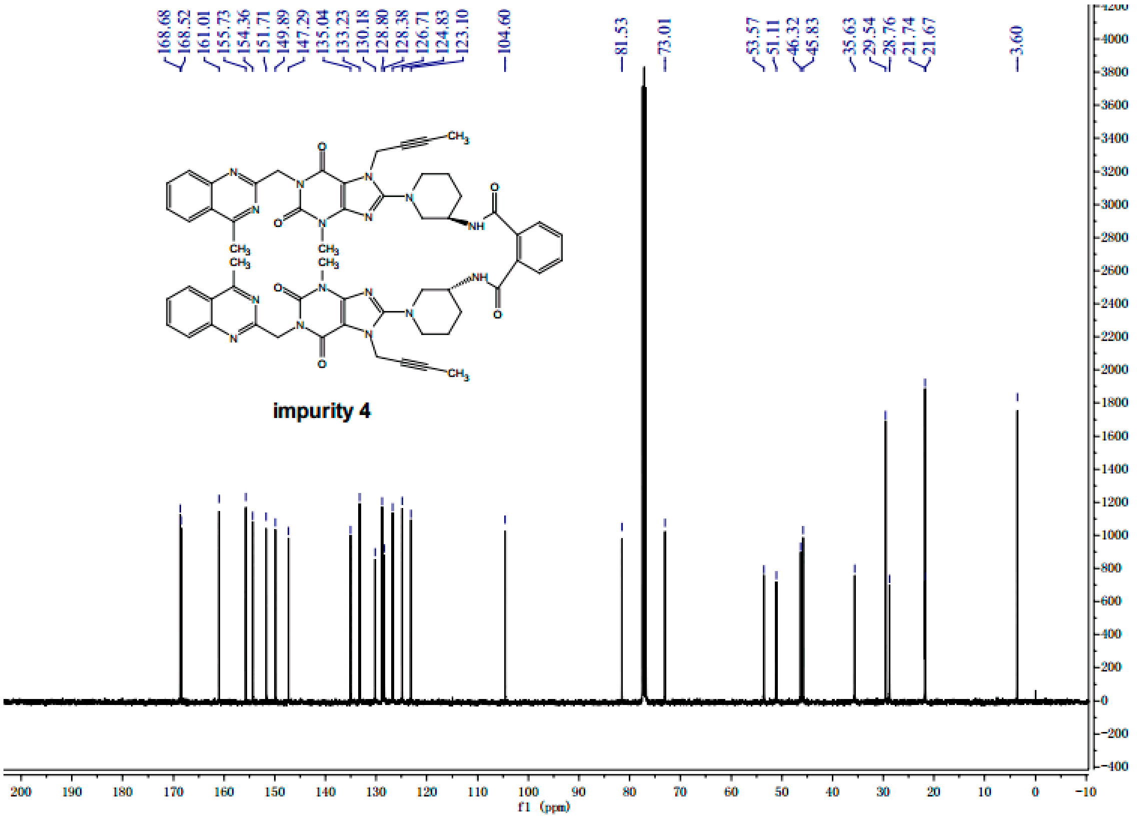

Figure S36. ^{13}C-NMR spectrogram of impurity **4**.

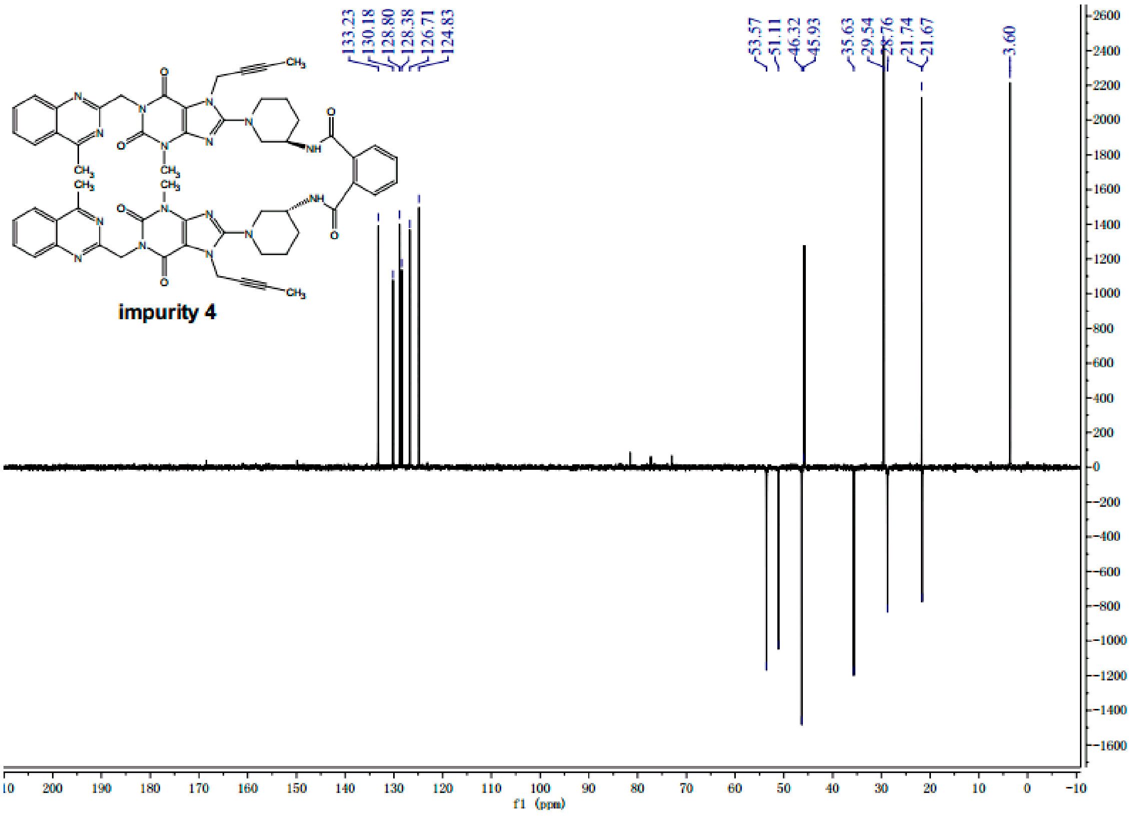

Figure S37. DEPT of impurity **4**.

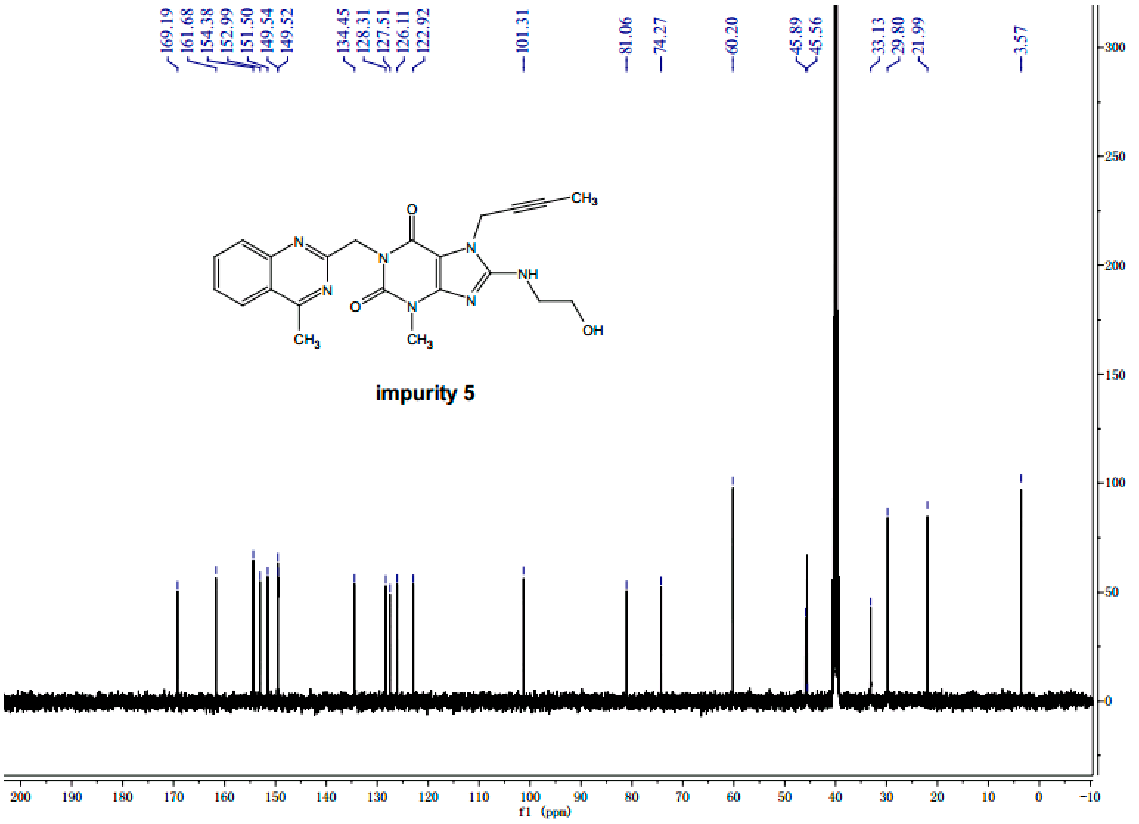

Figure S38. ^{13}C-NMR spectrogram of impurity **5**.

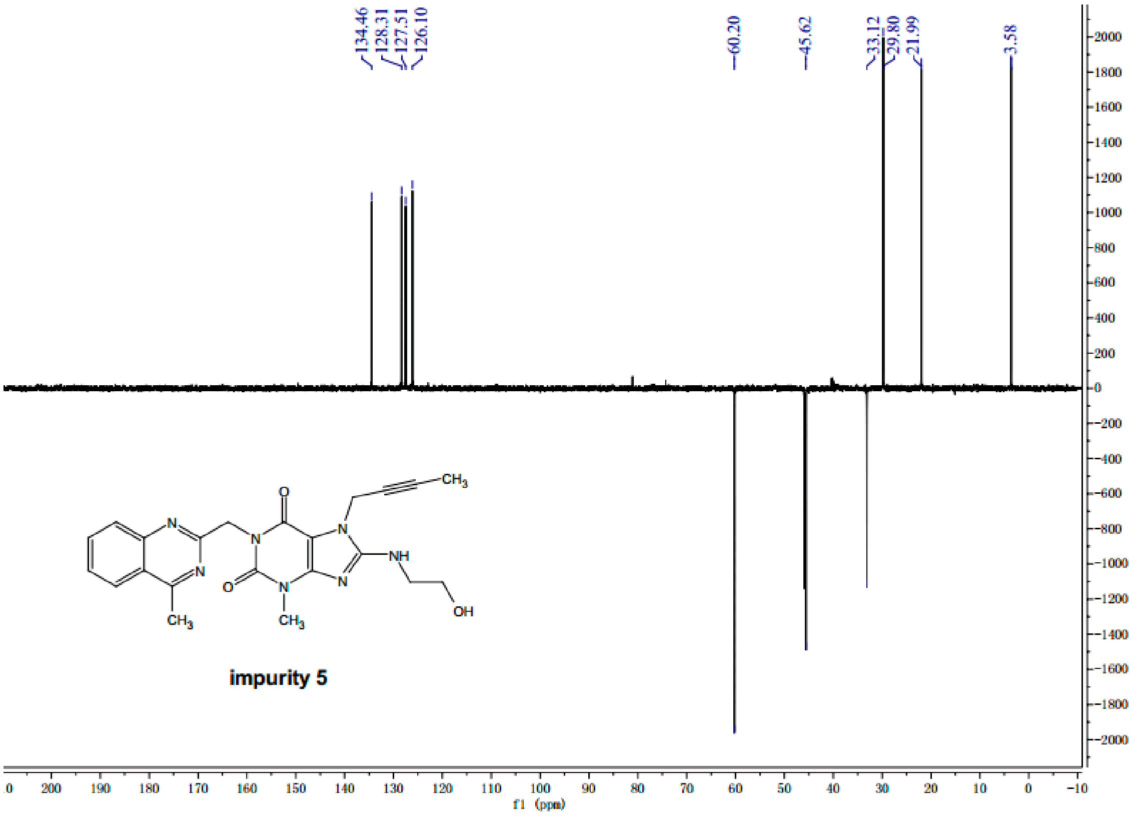

Figure S39. DEPT of impurity **5**.

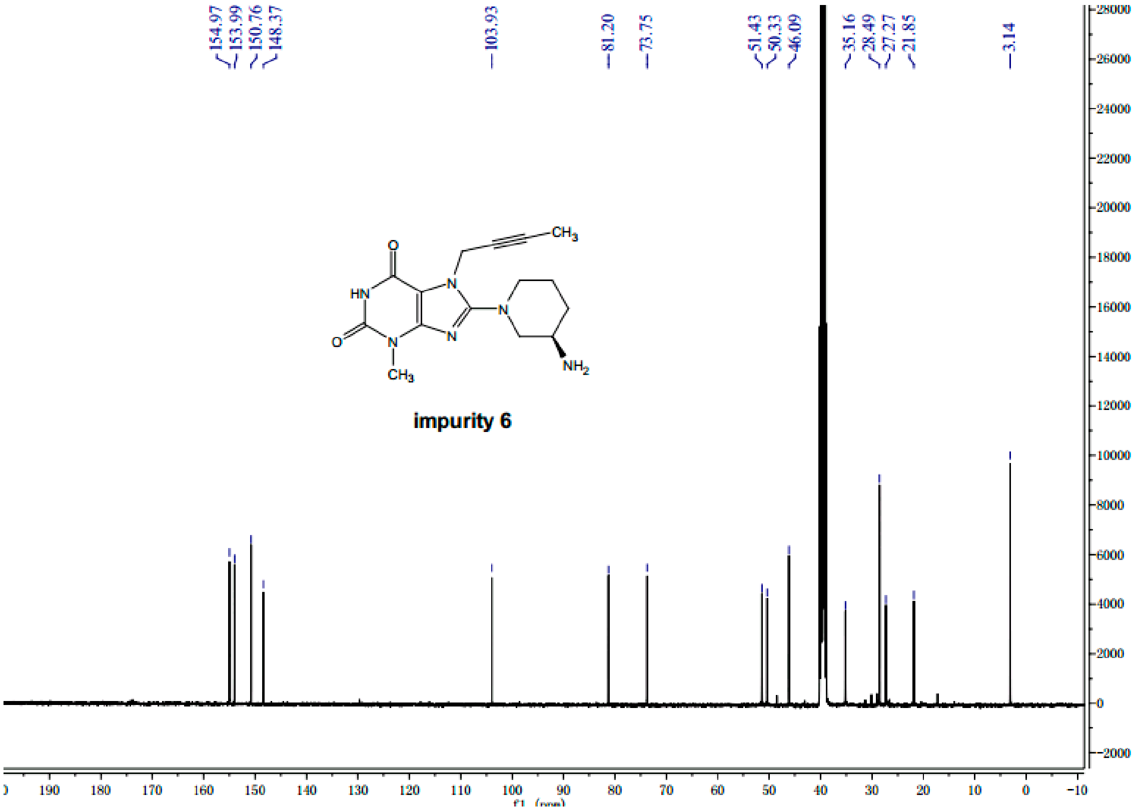

Figure S40. ^{13}C-NMR spectrogram of impurity **6**.

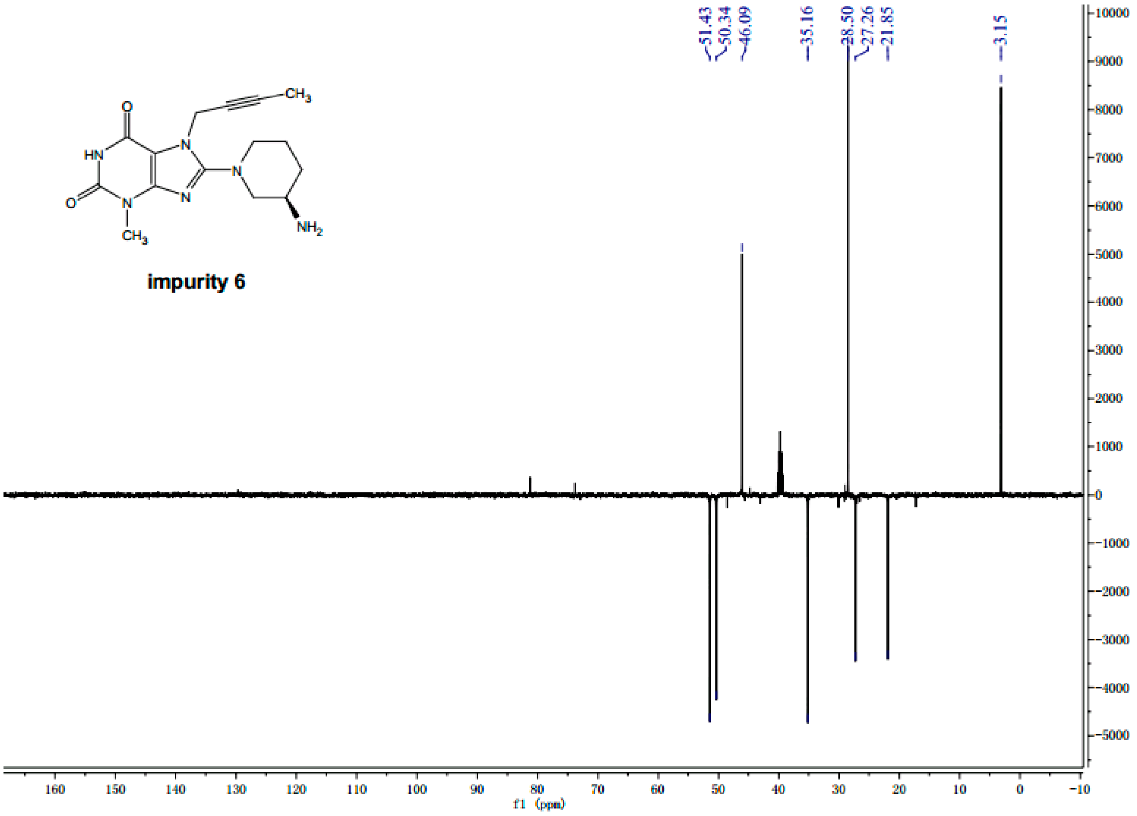

Figure S41. DEPT of impurity **6**.
